# Photoinduced crystal melting with luminescence evolution based on conformational isomerisation†

**DOI:** 10.1039/d3sc00838j

**Published:** 2023-04-20

**Authors:** Mao Komura, Hikaru Sotome, Hiroshi Miyasaka, Takuji Ogawa, Yosuke Tani

**Affiliations:** a Department of Chemistry, Graduate School of Science, Osaka University Toyonaka Osaka 560-0043 Japan y-tani@chem.sci.osaka-u.ac.jp; b Division of Frontier Materials Science and Centre for Advanced Interdisciplinary Research, Graduate School of Engineering Science, Osaka University Toyonaka Osaka 560-8531 Japan; c Innovative Catalysis Science Division, Institute for Open and Transdisciplinary Research Initiatives (ICS-OTRI), Osaka University Suita Osaka 565-0871 Japan

## Abstract

The phenomenon of crystal melting by light irradiation, known as photo-induced crystal-to-liquid transition (PCLT), can dramatically change material properties with high spatiotemporal resolution. However, the diversity of compounds exhibiting PCLT is severely limited, which hampers further functionalisation of PCLT-active materials and the fundamental understandings of PCLT. Here, we report on heteroaromatic 1,2-diketones as the new class of PCLT-active compounds, whose PCLT is based on conformational isomerisation. In particular, one of the diketones demonstrates luminescence evolution prior to crystal melting. Thus, the diketone crystal exhibits dynamic multistep changes in the luminescence colour and intensity during continuous ultraviolet irradiation. This luminescence evolution can be ascribed to the sequential PCLT processes of crystal loosening and conformational isomerisation before macroscopic melting. Single-crystal X-ray structural analysis, thermal analysis, and theoretical calculations of two PCLT-active and one inactive diketones revealed weaker intermolecular interactions for the PCLT-active crystals. In particular, we observed a characteristic packing motif for the PCLT-active crystals, consisting of an ordered layer of diketone core and a disordered layer of triisopropylsilyl moieties. Our results demonstrate the integration of photofunction with PCLT, provide fundamental insights into the melting process of molecular crystals, and will diversify the molecular design of PCLT-active materials beyond classical photochromic scaffolds such as azobenzenes.

## Abstract 2

UV irradiation induced conformational isomerisations of a 1,2-diketone in the crystal, causing multistep luminescence changes and eventual macroscopic melting.

## Graphical abstract

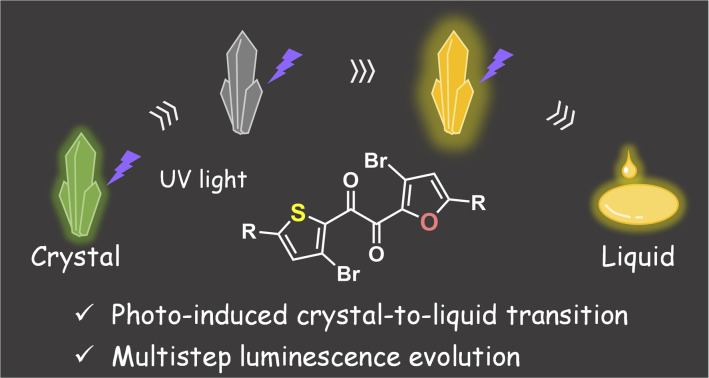

## Introduction

Photo-induced crystal-to-liquid transition (PCLT) is the phenomenon of crystal melting by light irradiation,^1–16^ in which photoexcitation causes molecular structural changes in the crystal and eventually leads to the melting. PCLT has been actively investigated due to a fundamental interest in the underlying mechanism/process of transducing light energy from the molecular structure change to macroscopic phase transition. Moreover, considerable effort has been focused on applications such as photolithography,^8^ thermal energy storage,^9–12^ and light-melt adhesion^13–15,17,18^ because PCLT can induce drastic changes in the macroscopic physical properties. However, the PCLT-active molecular motifs are severely limited. Namely, PCLT has been observed in only three photochromic motifs: azobenzene,^1–11,13–15^ hydrazone,^12^ and spiropyran^16^ that undergo *E*/*Z* isomerisation or the cleavage/formation of the σ-bond in crystals upon excitation. As a consequence, whether being molecularly photochromic is mandatory or not is inconclusive; a minimum requirement for the molecules to be PCLT-active can be to exhibit excited-state structural changes. Given that various photofunctional molecules other than photochromic ones exhibit considerable structural changes at the excited states, the molecular design and functions of PCLT-active materials hold immense potential for increased diversity. In particular, photofunctions that are sensitive to the molecular conformation and/or environment are worth integrating with PCLT; such functions will be useful to reveal how the molecular structural change leads to the melting of the whole crystal.

**Fig. 1:**
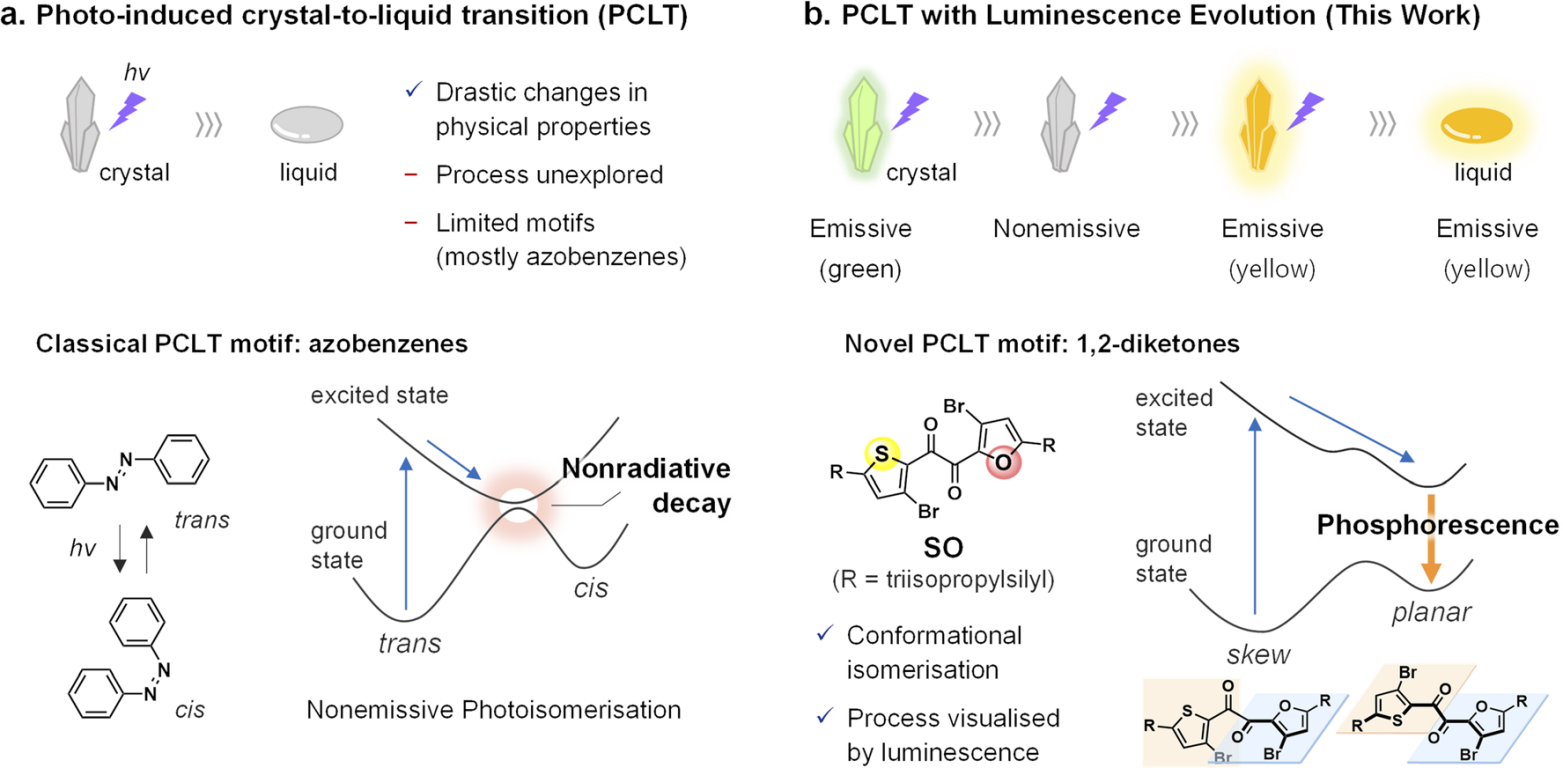
Schematic illustration of the photoinduced crystal-to-liquid transition (PCLT), chemical motifs, and their photophysical pathways. (a) Classical PCLT without luminescence properties. (b) PCLT with luminescence evolution. See Fig. S15 and S16† for calculated potential energy curves of SO.

Luminescence is a promising property for the *in situ* observations of phase transitions because it allows the real-time, high-sensitivity detection of the emissive part in a bulk material.^19–22^ Microscopic visualisations of the crystallisation process based on time-course luminescence have been reported previously.^23–27^ However, to the best of our knowledge, PCLT has never been studied using luminescence. Although azobenzenes are a promising motif for studying PCLT, their photoinduced *trans*–*cis* isomerisation dissipates the excited-state energy nonradiative (Fig. 1a).^28–30^

We had previously observed room-temperature phosphorescence (RTP) of a heteroaromatic diketone, SO (Fig. 1b).^31^ In contrast to typical metal-free organic phosphors that only phosphoresce in the crystal form,^32–36^SO exhibits RTP in its supercooled liquid (SCL) state (*i.e.*, the metastable liquid state at temperatures lower than its melting point). Detailed experimental and theoretical investigations revealed that SO exists in distinct rotational isomers, including skew and planar conformers (Fig. 1b). Most notably, while the crystal consists of the poorly emissive skew conformer, the planar conformer is more stable in the excited state and is responsible for the RTP in the SCL state.^31,37–40^ Such conformation-dependent RTP would be promising to visualise PCLT.

Here, we report on heteroaromatic 1,2-diketones as a novel class of PCLT-active compounds, which do not contain conventional photochromic frameworks. Moreover, one of the diketones, SO, exhibits PCLT accompanied by luminescence evolution (Fig. 1b). During light irradiation, the SO crystal exhibits two-step changes in the luminescence colour and intensity, which are reflective of the molecular conformation and environment and allow for the visualisation of the local melting process in real-time (Movies S1 and S2†). Although the photo-induced RTP enhancement of organic crystals has been actively investigated, none of them lead to the melting transition.^41–46^ Our work demonstrates the functional diversification of PCLT-active motifs, which expands future directions toward the development of PCLT-related materials.

## Results and discussion

### Real-time tracking of PCLT with luminescence evolution

**Fig. 2:**
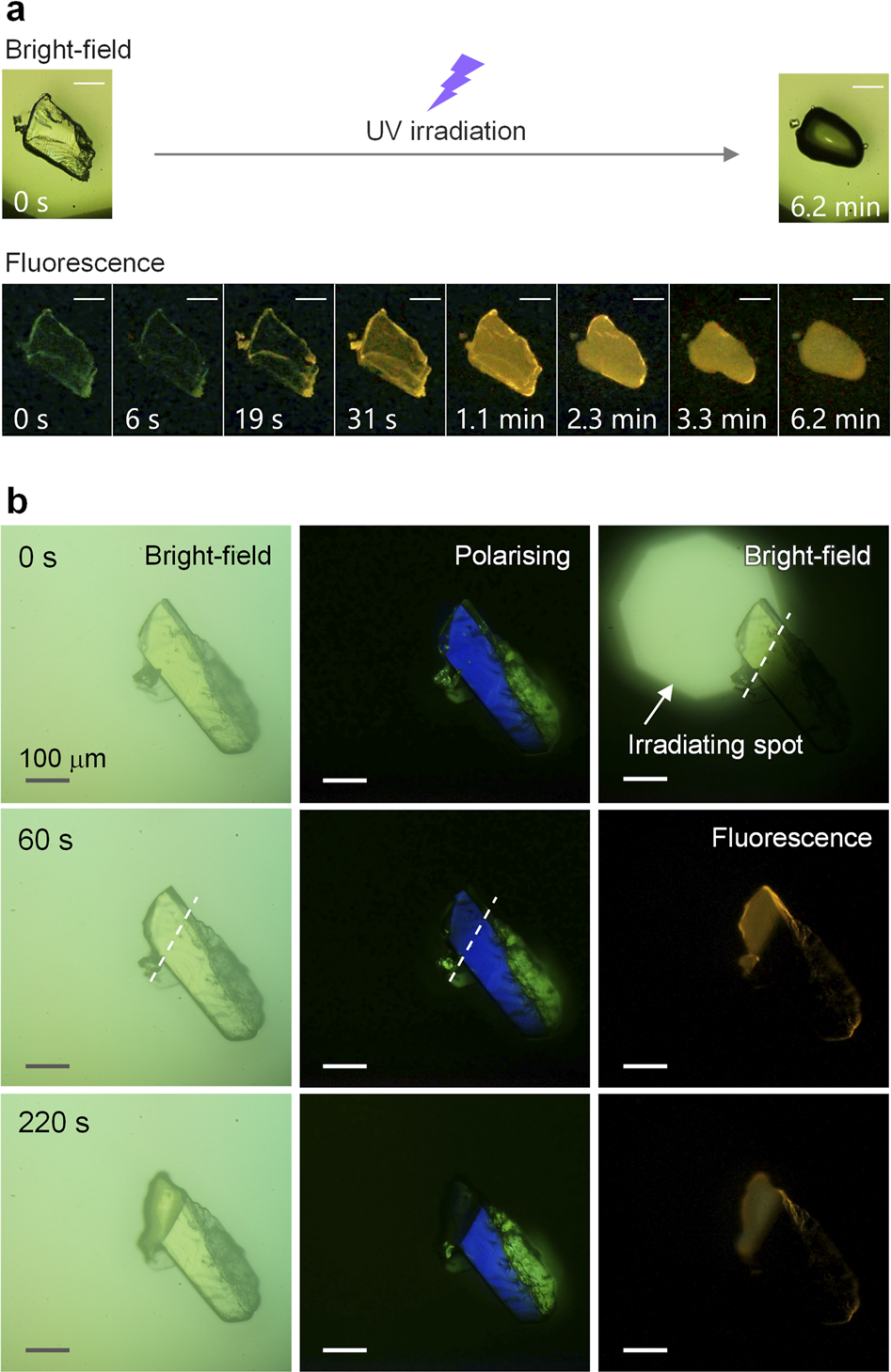
Photographic images of the photoinduced crystal melting with luminescence evolution observed under microscope. (a) Whole crystal was exposed to UV irradiation (scale bar: 50 μm). (b) Top-left part of the crystal was exposed to UV irradiation. Polarising optical microscope images were taken under cross Nichol prism.

Our research was initiated by an unexpected observation of the photoinduced melting of the SO crystal, accompanied by the changes in luminescence intensity and colour^41–46^ under a fluorescence microscope (Fig. 2a and Movies S1 and S2†). Under ultraviolet (UV) irradiation (60 mW cm^−2^, *λ*_max_ = 365 nm), the crystal initially exhibits a feeble green emission, which disappeared within few seconds. Moreover, the yellow emission, which is assigned as phosphorescence (Fig. S3†), emerged and expanded to the whole crystal. Finally, the crystal melted into an isotropic liquid, as evident from the disappearance of the birefringence (Fig. S4†). As reported previously, SO forms a kinetically super-stable SCL at room temperature that exhibits the same yellow RTP.^31^ Hence, its melting is a crystal-to-SCL transition, that is, a transition to the kinetically trapped metastable phase.

Next, to learn more about the photomelting phenomenon, UV light was irradiated on the top-left part of the crystal (Fig. 2b, top right). After 220 s, only the irradiated area exhibited yellow emission and melted (Fig. 2b, bottom row). The sharp boundary indicates that the melting transition does not propagate to the unirradiated crystal region and is not caused by heat. Note that the emergence of the yellow luminescence also supports the non-heat-induced melting, because the phosphorescence of the SCL state disappears at higher temperatures (lower than the melting point, which is 59.5 °C, see Fig. S5†). In addition, the sharp boundary also indicates that the recrystallisation of the melt is sufficiently slow, likely owing to the high kinetic stability of the SCL state. Most notably, the turning-on of the luminescence preceded the macroscopic melting. The bright-field and polarising optical images indicated that the irradiated area was still crystalline after 60 s irradiation, while a distinct emission was observed simultaneously (Fig. 2b, middle row). Thus, the emission is clearly indicative of molecular-level melting, which is not evident from the light reflection, transmission, and absorption characteristics.

**Fig. 3:**
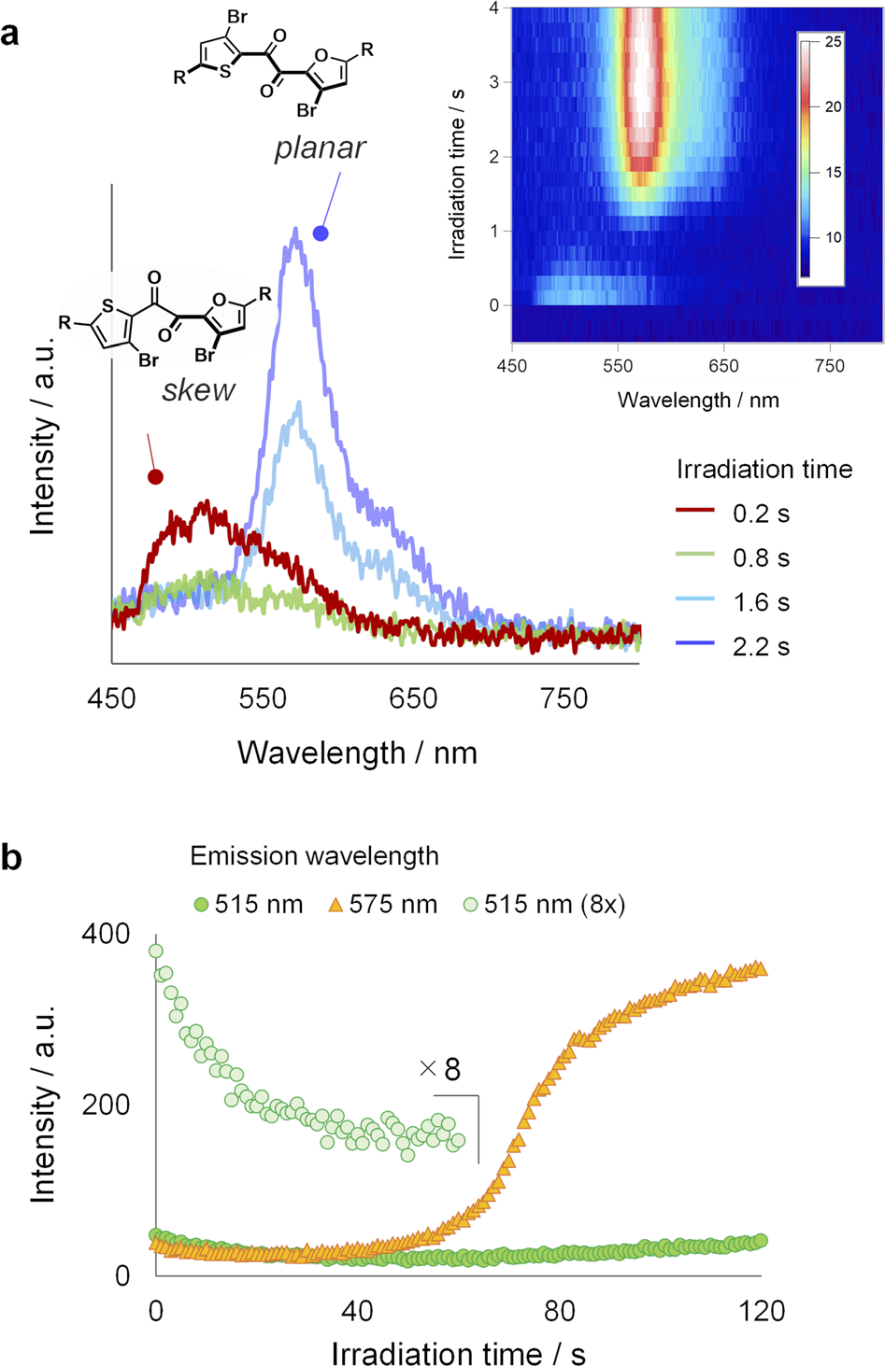
Time course of (a) photoluminescence spectra and (b) luminescence intensity (*λ*_em_ = 515 nm and 575 nm) of SO crystal during continuous UV irradiation. Note that crystal size and light source for panels (a) and (b) are different.

To gain further insights into the luminescence evolution during PCLT, we tracked the changes in the luminescence spectrum of a single crystal under continuous UV irradiation (Fig. 3a). UV excitation initially resulted in a weak green luminescence peak at approximately 520 nm, which disappeared within a second (Fig. 3a, red trace). The yellow luminescence (*λ*_max_ = 575 nm) turned on only after an inductive period. In another independent experiment, we observed that the yellow luminescence intensity increased in a sigmoidal manner as irradiation time advanced, indicative of the autocatalytic nature of the molecular process behind the luminescence evolution (Fig. 3b).^47–49^ The yellow emission matches the photoluminescence spectrum of the SCL state and can be ascribed to the RTP from the planar conformer (Fig. S4†).^31^ Meanwhile, the initial green emission is assignable to the skew conformer because SO exhibits the skew conformation in the crystal (see below, Fig. S10†).^31,37–39^ Therefore, the luminescence evolution reflects the local melting process, which involves the conformation change from skew to planar.^50–52^ Such conformation change are supported by Raman microscopy experiments (Fig. S19; see ESI† for details).

### Correlation between crystal structure and PCLT

**Fig. 4:**
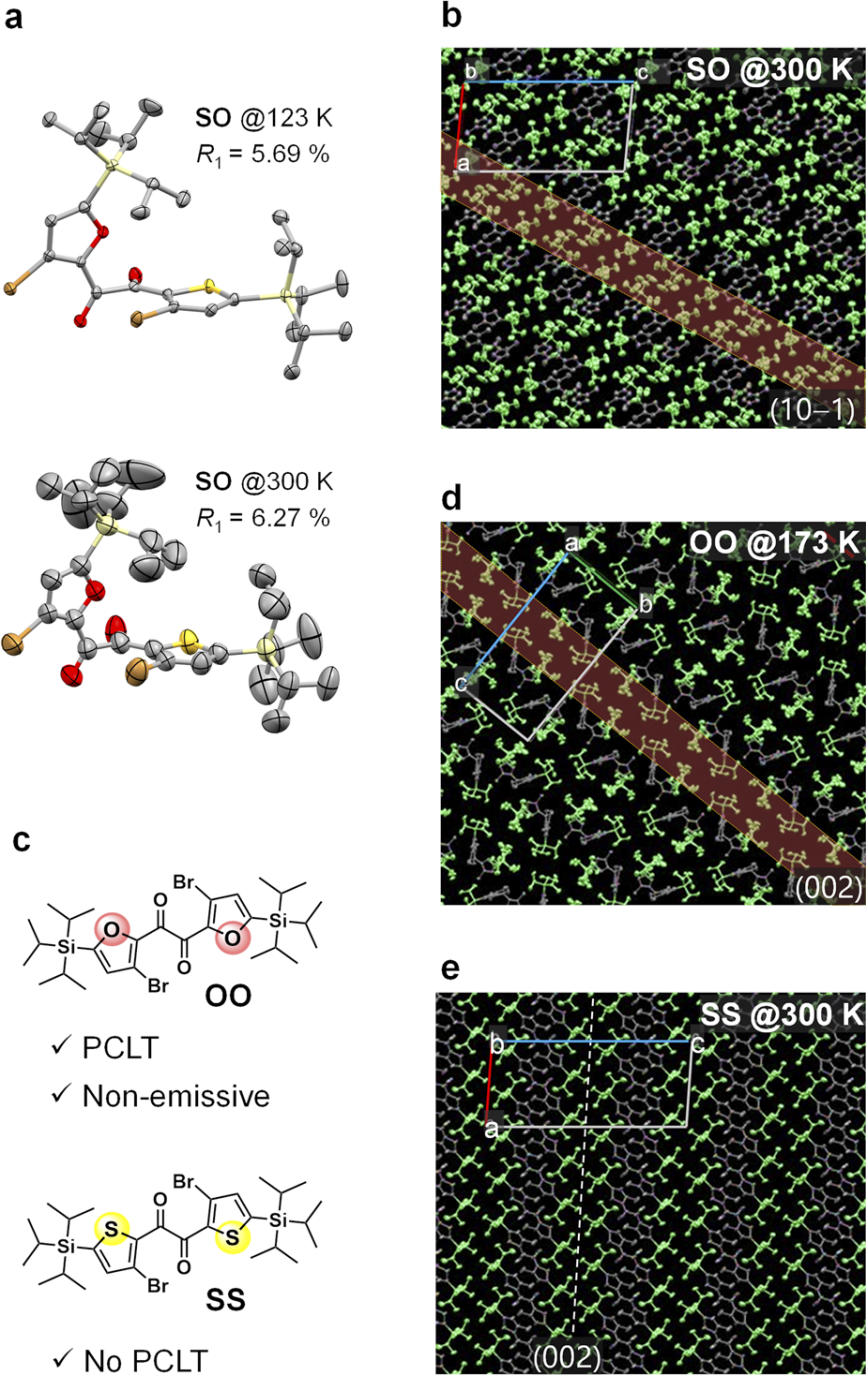
ORTEP drawings of (a) molecular structures of SO at 123 and 300 K and packing structure of (b) SO at 300 K, (d) OO at 173 K, and (e) SS at 300 K. Thermal ellipsoids are set at 50% probability level, and hydrogen atoms are omitted for clarity. In the panels (b), (d), and (e), the silyl moieties and diketone cores are drawn in green and grey, respectively. The disordered layer along the lattice plane facing silyl moieties are highlighted. (c) Chemical structures of OO and SS.

Although it is evident that the molecular conformation changes from skew to planar in the crystal, how such a large structural change proceeds is unclear. To elucidate the details of the large structural change, we investigated the crystal structure and examined its relaxation after UV excitation. We successfully performed a single-crystal X-ray structural analysis of SO at 300 K (Fig. 4a and S11†). Compared to the structure at 123 K,^31^ the thermal ellipsoids become large particularly in the silyl moieties. The packing structure shows that the silyl moieties form disordered layers along the (10−1) planes, while the heteroaromatic 1,2-diketone cores are relatively more ordered (Fig. 4b).^53^ Furthermore, according to density functional theory (DFT) calculations, the silyl moiety moves along the disordered (10−1) planes upon the excited-state structural relaxation, which is smaller than the skew-to-planar conformational change (Fig. S15 and S20†). Therefore, the mechanical stress in the crystal upon relaxation is relatively small and is mitigated by the disordered layer; thus, the relaxation is allowed, thereby accelerating the disordering of the entire crystal. The photoinduced loosening of the crystal was confirmed by comparing single-crystal X-ray structures before and after UV irradiation. Thus, the equivalent isotropic atomic displacement parameters (*U*_eq_), which represent the size of the thermal ellipsoid and hence the degree of disorder, became large by UV irradiation for 33 out of 36 atoms; the increase rates were up to 6% (Fig. S12†). In general, the RTP disappears when the ordered crystalline lattice is disturbed.^32–34^ Therefore, the disappearance of the skew emission is due to pronounced nonradiative decay and indicates crystal loosening owing to UV irradiation.^37^ Moreover, the sigmoidal increase of the planar emission intensity after the inductive period (Fig. 3b) indicates that the formation of the planar conformer is autocatalytic, and crystal loosening is necessary to overcome the barrier for it.^47–49^

To gain further insights into the factors controlling PCLT, we examined the photoresponse and crystal structures of the analogous diketones OO and SS (Fig. 4c). Despite the similarity in their chemical structures, with only one atom being different, their crystals responded differently upon UV irradiation; PCLT did not occur in the case of the SS crystal, while the OO crystal melted (without luminescence) (Fig. S21 and S22†). Single-crystal X-ray structural analysis revealed that OO exhibits packing features similar to those of SO, while SS does not (Fig. 4b, d and e). Thus, while all three diketones have a lattice plane facing the silyl moieties, in the cases of SO and OO, the two silyl moieties within a single molecule face the same plane, whereas in SS, they face a different plane. Moreover, the silyl moieties of SO and OO are disordered, while those of SS are well ordered even at room temperature (Fig. 4e and S23†). These results further confirm the importance of a crystal structure with a disordered layer for a molecular crystal to be PCLT-active.^5,53^

Energetically, the skew-to-planar photoisomerisation is favourable in all three diketones (Table S4†). However, the barrier to the structural change should increase if the intermolecular interactions in the crystals increase. Using differential scanning calorimetry, the enthalpy of fusion (Δ*H*_m_) values of the SO, OO, and SS crystals were determined to be 22, 29, and 40 kJ mol^−1^, respectively (Fig. S26–S28†). The values for SO and OO were significantly smaller, indicating that the intermolecular interactions in these PCLT-active crystals were weaker. The differences in Δ*H*_m_ reflect the different packing features, which is due to the different molecular conformations in the crystals (Fig. 4a, S23, and S24; see ESI† for detailed discussions). Moreover, the Gibbs free energies for the crystal-to-SCL transition at 300 K for SO, OO, and SS were estimated to be 2.2, 2.7, and 12 kJ mol^−1^, respectively. Clearly, PCLT is less endothermic in the cases of SO and OO. These results suggest that a subtle atomic replacement can significantly modulate the bulk thermal properties, resulting in PCLT in aromatic 1,2-diketones.

### A plausible PCLT mechanism of SO crystal

**Fig. 5:**
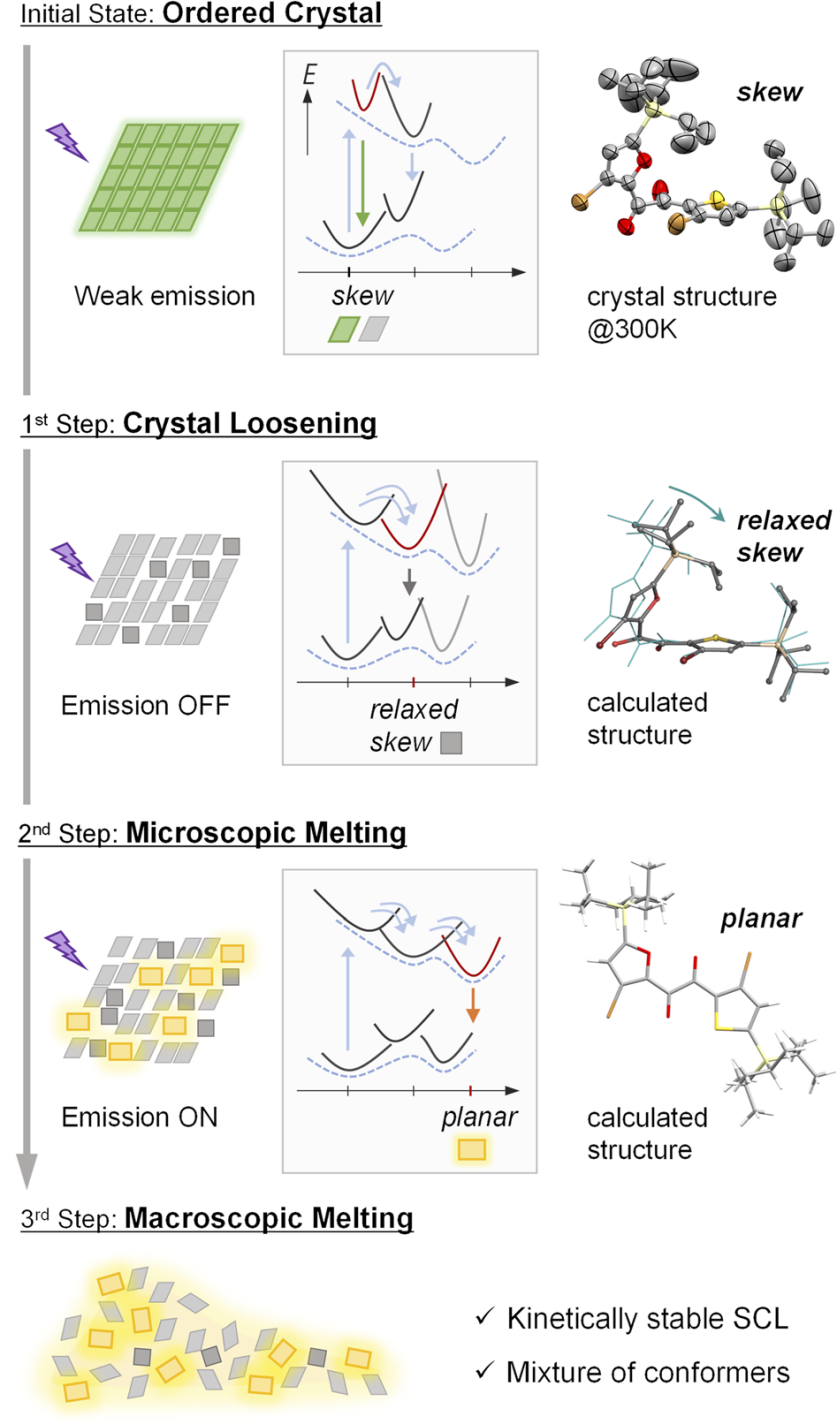
Schematic illustration of plausible PCLT mechanism. Left: the disordering crystal packing with luminescence properties. Panels on the middle: the potential energy curves, with solid lines for the molecules in crystal and broken lines for isolated molecules. Right: the corresponding molecular conformations.

Based on our results, a possible total three-step process for PCLT in the SO crystal is suggested (Fig. 5). Initially, the SO crystal exhibits a weak emission at 520 nm, which originates from the skew conformation. As the first step, the crystal packing is loosened because of the photoinduced conformational relaxation. The loosening is evident from the disappearance of the skew emission and single-crystal X-ray structure analyses. Meanwhile, as the second step, the loosening allows a larger conformational change to generate the planar conformer, providing the yellow RTP (microscopic melting). These events can be regarded as premelting, as they occur while the bulk substance is still solid. The presence of an inductive period and the sigmoidal response of the yellow RTP indicates that the skew-to-planar conformational change is impossible at first; the loosening of the crystal packing through the smaller conformational relaxation is necessary beforehand. These conformational changes further loosen the crystal packing and promote subsequent conformational changes, which occurs repeatedly in an autocatalytic manner, finally leading to greater disordering in the longer range and the eventual macroscopic melting of the bulk crystal (the third step). Thus, the two-step changes in the luminescence of the PCLT-active crystal allowed the visualisation of its molecular-level melting process in real-time.

It should be worth noting that, to induce PCLT, photoexcited molecules within the crystal must undergo a structural change. However, the dilemma exists that relatively small structural changes, which are comparatively easier to occur, rarely lead to phase transitions. Conversely, larger structural changes that could lead to phase transitions are hard to occur in the crystal. It would be this dilemma that limits the diversity of the PCLT-active compounds. In contrast, PCLT of the diketones occurred not in one step but involved two types of structural changes that induce disordering. Such a PCLT mechanism can avoid the dilemma and diversify the molecular design of PCLT-active materials.

The proposed PCLT mechanism is based on a conformational change around the single bonds. Meanwhile, the reported mechanisms for the PCLT of photochromic molecules are based on the double-bond *E*/*Z* isomerisation^1–12^ or the cleavage/formation of the σ-bond.^16^ These photoreactions have a high energy barrier for the thermal back reaction in the ground state, so that those conventional motifs are photochromic as an isolated molecule. In contrast, the barrier for the backward reaction of SO is not high (calculated to be 6.86 kcal mol^−1^ in vacuum, Fig. S16†). Nonetheless, the SCL state of SO exhibits exceptionally high kinetic stability (greater than 3 months). We had previously demonstrated that SCLs do not crystallise readily even in the presence of crystal seeds.^31^ This is likely because the diketone core has at least eight conformers, including axially chiral enantiomers (Fig. S13†). Moreover, the zero-shear viscosity of SO SCL was 45 ± 2 Pa s at room temperature, which is four orders of magnitude thicker than conventional organic solvents.^31^ Such a highly viscous environment slows down the conformation changes and the translational and rotational motions of the molecules, resulting in the slow kinetics of assembling and aligning molecules to form crystals. Thus, the backward recrystallisation process is extremely slow to compete with the melting process. Indeed, even at the earliest stage of the PCLT, it is virtually irreversible within a reasonable time range. The photoluminescence spectra did not change after keeping the specimen in the dark for 20 min after ceasing UV irradiation at the first step of PCLT (Fig. S9†). The elucidation of this mechanism should help improve the molecular design of PCLT-active materials and lead to the development of materials other than the conventional photochromic motifs.

## Conclusions

In summary, we present the photoinduced crystal-melt transition (PCLT) of heteroaromatic 1,2-diketones. In particular, diketone SO exhibits PCLT accompanied by luminescence evolution. Thus, the dynamic multistep changes in the luminescence colour and intensity visualised the PCLT process involving two-step conformation changes, which furthers our understanding of the melting phenomenon at the molecular level. Based on the results and comparisons with the other two diketone derivatives, we clarified that the presence of a disordered layer in a crystal is an important factor for PCLT in diketone scaffolds. It should be noted that unlike the PCLT of conventional photochromic motifs, such as azobenzenes, the diketone demonstrates PCLT based on conformational isomerisation, *i.e.*, rotation around single bonds. Considering that various photofunctional molecules are not photochromic as a single molecule but exhibit considerable structural changes at the excited states, our work should lead to the diversification and multifunctionalisation of PCLT-active motifs.

## Data availability

All experimental/computational procedures and data related to this article are provided in the ESI.†

## Author contributions

M. K. conceptualisation: equal; investigation: lead; visualisation: lead; writing—original draft: lead; writing—review & editing: equal. H. S. conceptualisation: supporting; funding acquisition: supporting; investigation: supporting; visualisation: supporting; writing—review & editing: supporting. H. M. resources: supporting; writing—review & editing: supporting. T. O. conceptualisation: supporting; resources: lead; supervision: supporting; writing—review & editing: supporting. Y. T. project administration: lead; supervision: lead; conceptualisation: equal; funding acquisition: lead; investigation: supporting; visualisation: supporting; writing—original draft: supporting; writing—review & editing: lead.

## Conflicts of interest

There are no conflicts to declare.

## Supplementary Material

SC-014-D3SC00838J-s001

SC-014-D3SC00838J-s002

SC-014-D3SC00838J-s003

SC-014-D3SC00838J-s004

SC-014-D3SC00838J-s005
