# Supplementary material for: Photoinduced crystal melting with luminescence evolution based on conformational isomerisation
Source: Chem Sci. 2023 Apr 20;14(20):5302–8. doi: 10.1039/d3sc00838j (PMC10207888; doi:10.1039/d3sc00838j)
Supplement: SC-014-D3SC00838J-s001 [file SC-014-D3SC00838J-s001.pdf]

Electronic Supplementary Material (ESI) for Chemical Science.
This journal is © The Royal Society of Chemistry 2023

SO-planar (opt.@T_1)

E(UB3LYP) = -7445.03528619 Hartree

C	-4.212067646297	-0.192067058886	-0.980822384758
C	-4.152147714944	-0.433474920034	-2.329754706701
H	-4.988032164546	-0.557388852418	-3.000741434041
C	-2.777410552651	-0.492108223522	-2.682276663530
C	-2.053976113263	-0.279940314155	-1.524710870898
C	-0.628817395586	-0.221066040869	-1.286381361682
O	0.193234389567	-0.403918229309	-2.194243902113
Br	-2.106992508275	-0.800260798683	-4.411700696400
Si	-5.582977370549	0.052896399221	0.295899037537
C	-5.117379485117	-1.040536207850	1.782529847725
H	-4.187086010414	-0.583773171647	2.146336933414
C	-4.783480947171	-2.499290874432	1.425730067893
H	-4.400612795709	-3.029863965434	2.304601383756
H	-4.018703628389	-2.564782844894	0.647705210911
H	-5.661121870761	-3.048354585391	1.075538115365
C	-6.153245031855	-0.967999505722	2.918295128333
H	-7.097181503733	-1.442135437996	2.631530409625
H	-6.378054056972	0.060857107421	3.215534124398
H	-5.790807132679	-1.491662845304	3.809707534790
C	-5.559810923281	1.889627427436	0.805422265986
H	-6.415603624997	2.031688097661	1.480312045740
C	-4.283053867827	2.272203245825	1.577139810084
H	-3.377998003439	2.069200570141	0.998410072085
H	-4.190854703201	1.726261589221	2.519529974523
H	-4.285693761336	3.340684210243	1.820222169795
C	-5.760065129277	2.811647881558	-0.411014582150
H	-5.792236905183	3.862463237784	-0.103082029870
H	-6.689868198969	2.602185254372	-0.948295528608
H	-4.937575010954	2.708712837754	-1.125865333754
C	-7.210323691172	-0.355742563804	-0.608191602032
H	-7.134894721603	0.196635449272	-1.556468735182
C	-8.457274229663	0.170740391488	0.126290345527
H	-9.361807652205	-0.020385615375	-0.461670904412

H	-8.410461138564	1.247212714812	0.309557258948
H	-8.594291855610	-0.321573411533	1.093636590682
C	-7.375656592287	-1.846455962254	-0.954357298683
H	-7.553429826388	-2.444417907500	-0.056283745863
H	-6.497774382338	-2.262453039318	-1.455999096362
H	-8.236723403111	-1.998108783437	-1.614741353045
C	3.811173859187	0.126916301257	0.255174903228
C	3.310902615516	0.385168444829	1.513187278500
H	3.925992729770	0.572418890803	2.383924561186
C	1.901965545426	0.389590004967	1.565439998847
C	1.283073372420	0.131283628074	0.345467025835
C	-0.135982510262	0.077460763619	0.108129969641
O	-0.962946401627	0.276747841864	1.038607926647
Br	0.980212997384	0.731287341069	3.192331349723
Si	5.623215465995	-0.037618312458	-0.257432588625
C	6.634304129502	0.904606894934	1.058104676323
H	6.317874849055	0.444786113286	2.005638660938
C	6.319756179557	2.407744774257	1.153941765689
H	6.790812263227	2.845028266541	2.041423584288
H	5.247245285552	2.607650845093	1.216710096860
H	6.704388196729	2.952127527792	0.288078840199
C	8.151073316625	0.672117838465	0.931526705864
H	8.550883133750	1.113748943346	0.014142559787
H	8.413836890627	-0.389108267363	0.927694826228
H	8.684709195611	1.136473120078	1.768255403453
C	6.088350593640	-1.887885809046	-0.191090111982
H	7.152445991672	-1.941393334469	-0.460354328949
C	5.929354710261	-2.467689099760	1.225154289407
H	4.891200389359	-2.402381059100	1.566078588997
H	6.552248356800	-1.950480804621	1.960575895499
H	6.212374455474	-3.525869764338	1.248176636976
C	5.303347898361	-2.729878575555	-1.211812200292
H	5.616280773068	-3.779163283053	-1.172268141673
H	5.451281400596	-2.386841572453	-2.239494463579
H	4.228519513734	-2.707042818592	-1.008829557889
C	5.755456271989	0.580673470129	-2.055863797024

H	5.016107407584	-0.031159891161	-2.593745611243
C	7.126970519903	0.298433311665	-2.695250244913
H	7.118622835664	0.558070023752	-3.759537515567
H	7.417399744915	-0.752989088288	-2.619581342498
H	7.917976580025	0.892901124474	-2.228555885011
C	5.351306148096	2.051816030953	-2.255960985941
H	6.093867630661	2.732752923269	-1.832354538784
H	4.387419529647	2.285537346670	-1.797294031425
H	5.271900919570	2.287316544901	-3.322896826282
O	-2.926671720098	-0.101214213970	-0.498202948796
S	2.514006095786	-0.117197676306	-0.884566520672

SO relaxed skew (opt.@T_1)

E(UB3LYP) = -7445.02425346 Hartree

Br	9.476622367537	6.177483621916	5.045950100711
Br	8.175830991881	7.327022503614	1.172212180107
S	7.122942851726	7.971061475863	8.342768516834
Si	4.763698171478	3.780897735014	4.419608012588
Si	8.329401874434	6.254200980775	10.745465081388
O	5.949892611967	6.323633469903	4.419699765657
O	6.787875414053	9.905119864200	6.154430991415
O	6.926526396268	9.395828305880	3.387608788153
C	6.737815588432	7.197480050593	3.738830586856
C	7.099772781286	8.794169905356	5.728141806754
C	8.513003301459	6.727258765659	6.600625779793
C	7.036768222021	6.629967749959	2.482379229719
C	7.599872992716	7.759204469123	6.672397634020
C	6.454300084215	5.350808419930	2.461681477145
H	6.529209462061	4.629581875377	1.662923701010
C	8.806713282687	6.115351474233	7.848162342242
H	9.524850814804	5.310885457351	7.941617300014
C	7.015119525959	8.489670942359	4.293274608683
C	7.086154487814	4.881184716238	11.199568152370
H	7.204442083016	4.703406382759	12.277046961305
C	8.134687020899	6.676225524610	8.909768481423

C	4.944099424477	2.304082820994	3.227487802698
H	6.016419903459	2.279396823946	2.985588702898
C	5.792925847981	5.175140819566	3.658216085598
C	10.101322789922	5.574947680056	10.945281484945
H	10.120952993837	4.705112227698	10.272252365183
C	5.522862908324	3.374213622993	6.115043494302
H	4.970266498854	2.496290018545	6.478795041971
C	7.396696317970	3.560095687707	10.474550872194
H	7.426482863074	3.690727097279	9.389120052863
H	6.632431409739	2.804522270136	10.689143761772
H	8.359765677569	3.141677810237	10.779546446149
C	7.901805089461	7.831300719055	11.729557590677
H	6.930026895729	8.143223105429	11.318558062546
C	4.595739358544	0.956698197171	3.888775359799
H	5.194853206355	0.761730049755	4.781435355415
H	4.771883851640	0.129950779762	3.192090470206
H	3.542935541585	0.909432363110	4.182082267112
C	4.170742343427	2.465021915590	1.905664355805
H	3.091550492810	2.399638243744	2.068715167009
H	4.436564825997	1.668242438346	1.202568854085
H	4.365917063976	3.419425065406	1.409284423532
C	5.634373056547	5.332102983321	10.958086678609
H	5.367634148735	6.200993166067	11.566288796362
H	4.928305588398	4.532225173637	11.208245262467
H	5.461690999659	5.601829210509	9.913056988293
C	10.379488932723	5.052606943062	12.366814090034
H	10.407911953266	5.869041305367	13.094336766440
H	11.352411040654	4.550910681604	12.412734920721
H	9.627240144656	4.335248446987	12.706771485561
C	7.691954311886	7.574765931406	13.233376248056
H	6.939682008868	6.806049508046	13.428316014601
H	7.359885805546	8.489202243316	13.737064765349
H	8.618275235951	7.259860237207	13.722799559164
C	5.338056644002	4.502504632973	7.144780329927
H	5.856131857694	5.415826764495	6.844315429977
H	5.749153400897	4.200844986974	8.109823349896

H	4.286781745293	4.752423589274	7.308919544808
C	3.001817197155	4.476055560156	4.635840662697
H	3.118876996616	5.236102532478	5.419574974027
C	11.219267229281	6.527659995434	10.486773384949
H	11.041437211520	6.927855933818	9.486021215259
H	12.184074217723	6.008393397208	10.469201889650
H	11.326263941622	7.377398810090	11.164930899279
C	7.008308969391	2.985521301565	5.997519265585
H	7.172572369945	2.152449866107	5.307677546359
H	7.407856004968	2.684667436247	6.971901821786
H	7.612469018742	3.827030788676	5.648183016875
C	8.881502691259	8.998359344003	11.512327906571
H	9.841766415195	8.806255286471	11.998245798188
H	8.482428094808	9.921780850445	11.945683365152
H	9.076969835714	9.192689138835	10.455195513591
C	2.433473807091	5.193673169045	3.399320661985
H	3.098631759032	5.983533530689	3.040408444885
H	1.471898888733	5.661006157223	3.637787574592
H	2.258990080992	4.506206237914	2.568241804284
C	2.023106726082	3.410228961522	5.161492100106
H	1.832783297438	2.635498533534	4.412386508957
H	1.055651705028	3.860836055210	5.407932971079
H	2.389131035453	2.913747861854	6.065302928932

SO’ TS (opt.@S_0)

E(RB3LYP) = -6155.73967641 Hartree

C	-3.979590284703	1.847995486711	0.608827751456
C	-3.891115598593	0.528543176788	0.937371120008
C	-2.765539543606	-0.089619862632	0.320031159685
C	-2.023179129616	0.750107940876	-0.461966849688
S	-2.694471990857	2.351642357260	-0.438084384189
C	-0.800360766921	0.391164549431	-1.251710717476
C	0.542646553279	0.690241938342	-0.554606065525
O	-0.832954149041	-0.018530018955	-2.382817979116
C	1.553906810884	-0.351087880028	-0.545947012879

O	0.708318655043	1.803115496421	-0.092441778125
C	2.857718365721	-0.391827592538	-0.100703288513
C	3.334830799063	-1.713559173202	-0.312656861047
C	2.289473663009	-2.391821074239	-0.867261672545
O	1.214439176412	-1.595797181610	-1.015983612660
Br	3.851850909482	1.011338112799	0.648978191760
Br	-2.329523392704	-1.925775366954	0.585394135391
H	-4.724316758151	2.563595356732	0.925883439337
H	-4.583079431755	0.005950821080	1.583196012928
H	4.317197068714	-2.092701444241	-0.080815847214
H	2.167485044340	-3.410848642041	-1.198310741588

SO’ TS (opt.@T_1)

E(UB3LYP) = -6155.66692958 Hartree

C	-3.876113058093	-2.017732495193	0.052225014229
C	-3.966592328446	-0.799068064725	-0.562076924541
C	-2.865165475950	0.035522516056	-0.241136031839
C	-1.932002010041	-0.561660306941	0.576403309853
S	-2.451372098677	-2.169264864571	1.017333481320
C	-0.684666173963	0.030895579573	1.122586476354
C	0.539358928456	-0.284213721356	0.379479140436
O	-0.789550823794	0.736750771685	2.140737101576
C	1.816377204698	0.245161103893	0.802998289471
O	0.430729099681	-1.017022666494	-0.632428130838
C	3.083032530131	0.113385152424	0.276438201445
C	3.964717696261	0.859015073247	1.109922719795
C	3.179515645422	1.392557146334	2.084860568598
O	1.883452043894	1.033334873365	1.916265817451
Br	3.582528886570	-0.858034579224	-1.252780994329
Br	-2.680932112378	1.795811823167	-0.913537010465
H	-4.561113732213	-2.849307145708	-0.031530450588
H	-4.771072671615	-0.504099685005	-1.221361881734
H	5.030001078769	0.972382199682	0.987276481715
H	3.380249111289	2.023500599790	2.935316962091

OO-planar (opt.@T_1)

E(UB3LYP) = -7122.04349584 Hartree

C	-3.741124199355	-0.414843313181	-0.832666826307
C	-3.491517601963	-0.864871147732	-2.104330891972
H	-4.224434013889	-1.142148640369	-2.846042164007
C	-2.081578450810	-0.898865891439	-2.263440985630
C	-1.528696925034	-0.460360743065	-1.073672857648
C	-0.148715480469	-0.282782827813	-0.688613089275
O	0.775121959747	-0.580406619367	-1.468166820880
Br	-1.163073067898	-1.438923979386	-3.813395486832
Si	-5.279932982566	-0.054758769855	0.201793264739
C	-5.040800153551	-0.976117771910	1.850190806049
H	-4.174294477733	-0.468495617988	2.295066355267
C	-4.660303741969	-2.459770490558	1.707220245576
H	-4.408557866175	-2.886216023045	2.684656591714
H	-3.791968798665	-2.598603970585	1.057990108435
H	-5.478959764750	-3.056546025932	1.297293951363
C	-6.233303264457	-0.796502676973	2.806252435555
H	-7.125816513347	-1.310913176500	2.435986416397
H	-6.496463912398	0.255290547831	2.954331371632
H	-6.008558227950	-1.216889563672	3.792679703071
C	-5.328642513136	1.825597652786	0.509273365695
H	-6.284977239813	2.028680488659	1.011526112591
C	-4.197838536078	2.304668430081	1.439093640804
H	-3.208706723706	2.049016748867	1.050495885883
H	-4.273244086212	1.867489950967	2.438147983447
H	-4.234533657041	3.393380023643	1.558775774133
C	-5.316682932331	2.609566156736	-0.815617901158
H	-5.400647196508	3.686395534211	-0.632560356517
H	-6.140323764377	2.327420693624	-1.478430179347
H	-4.383645947441	2.445984469530	-1.363906740782
C	-6.758842894212	-0.583120212638	-0.879129231240
H	-6.537713633825	-0.148282998091	-1.864932997961
C	-8.096661007168	0.016261022065	-0.407546451784
H	-8.906148964386	-0.257488454690	-1.093340252857

H	-8.069726587460	1.107633945325	-0.356167970240
H	-8.377923744283	-0.352654097285	0.583307515743
C	-6.885216464447	-2.106231095068	-1.063613359820
H	-7.203645187181	-2.592118962105	-0.137206393963
H	-5.945962101506	-2.573727111853	-1.371247639771
H	-7.637589506219	-2.343983951633	-1.823889405704
C	3.741124199355	0.414843313181	0.832666826307
C	3.491517601963	0.864871147732	2.104330891972
H	4.224434013889	1.142148640369	2.846042164007
C	2.081578450809	0.898865891439	2.263440985630
C	1.528696925034	0.460360743065	1.073672857648
C	0.148715480469	0.282782827813	0.688613089275
O	-0.775121959747	0.580406619367	1.468166820880
Br	1.163073067898	1.438923979386	3.813395486832
Si	5.279932982566	0.054758769855	-0.201793264739
C	6.758842894212	0.583120212638	0.879129231240
H	6.537713633825	0.148282998091	1.864932997961
C	6.885216464447	2.106231095068	1.063613359820
H	7.637589506219	2.343983951633	1.823889405704
H	5.945962101506	2.573727111853	1.371247639771
H	7.203645187181	2.592118962105	0.137206393963
C	8.096661007168	-0.016261022065	0.407546451784
H	8.377923744283	0.352654097285	-0.583307515743
H	8.069726587460	-1.107633945325	0.356167970240
H	8.906148964386	0.257488454690	1.093340252857
C	5.328642513136	-1.825597652786	-0.509273365695
H	6.284977239813	-2.028680488659	-1.011526112591
C	5.316682932331	-2.609566156736	0.815617901158
H	4.383645947441	-2.445984469530	1.363906740782
H	6.140323764377	-2.327420693624	1.478430179347
H	5.400647196508	-3.686395534211	0.632560356517
C	4.197838536078	-2.304668430081	-1.439093640804
H	4.234533657041	-3.393380023643	-1.558775774133
H	4.273244086212	-1.867489950967	-2.438147983447
H	3.208706723706	-2.049016748867	-1.050495885883
C	5.040800153551	0.976117771910	-1.850190806049

H	4.174294477733	0.468495617988	-2.295066355267
C	6.233303264457	0.796502676973	-2.806252435555
H	6.008558227950	1.216889563672	-3.792679703071
H	6.496463912398	-0.255290547831	-2.954331371632
H	7.125816513347	1.310913176500	-2.435986416397
C	4.660303741969	2.459770490558	-1.707220245576
H	5.478959764750	3.056546025932	-1.297293951363
H	3.791968798665	2.598603970585	-1.057990108435
H	4.408557866175	2.886216023045	-2.684656591714
O	-2.536604461279	-0.172208278715	-0.210745240689
O	2.536604461279	0.172208278715	0.210745240689

OO-skew (opt.@T_1)

E(UB3LYP) = -7122.03659955 Hartree

Br	13.351439765710	7.263791725885	23.096690901303
Br	9.320322323820	9.788654240988	22.086633330628
Si	8.178397692418	12.381012373770	16.895367980191
Si	10.033503596360	6.089615612180	18.353734694446
O	11.381262679839	8.177513102281	19.663818817018
O	10.261506283061	11.505009844978	18.522150786654
O	13.509579795348	10.140982109702	21.727974153705
O	12.783146370198	11.830476769174	19.547053583522
C	12.131927574601	8.522808126580	20.749856386171
C	8.890249931867	11.503567465675	18.419127473205
C	10.622602337324	10.920723871967	19.701294403575
C	12.055861405563	10.951170978495	19.992930767446
C	12.583846858811	9.873077232547	20.918964986173
C	8.375542955496	10.923788639894	19.550128873281
H	7.333652884440	10.783618130809	19.791859831420
C	6.588588310504	11.487032546236	16.314990336331
H	5.768642023911	11.946570173383	16.883692322766
C	11.109249552882	6.824987377467	19.723122349356
C	9.476572525660	10.557733971075	20.371921505040
C	12.380411563246	7.377777067218	21.492256730834
C	11.714764936113	6.309331417612	20.842460515110

H	11.692824450805	5.280786321301	21.168415105895
C	7.783580050621	14.164111702832	17.445660931951
H	8.742567851985	14.562183903704	17.803904482495
C	9.529686635686	12.376998407451	15.555217876701
H	9.037064191415	12.762276084768	14.651533613768
C	6.792046367200	14.222020719554	18.619795805161
H	5.804130454703	13.844735130844	18.334610047899
H	6.651607780096	15.253833683994	18.960092612827
H	7.136575651324	13.639648670570	19.478585272932
C	6.555743729279	9.976041288575	16.601517147277
H	7.384539631680	9.449299674158	16.121123947482
H	5.628184390762	9.530605923282	16.224096549586
H	6.611512264553	9.757438455923	17.668444192607
C	10.709647148902	13.314293986457	15.878447235817
H	10.388329279796	14.348569849201	16.028991570705
H	11.430366679947	13.317254519006	15.053067727498
H	11.243162969369	12.996024455008	16.776411233442
C	8.252252089347	5.929035887162	19.015438080478
H	7.660274194937	5.509659381120	18.189943856299
C	10.035953063796	10.956794931603	15.243753454502
H	10.524840067504	10.515143742734	16.114779382890
H	10.771728726702	10.975528786412	14.432147680166
H	9.231932024473	10.282892858418	14.935692648602
C	7.307273405693	15.054459303521	16.283986923518
H	7.998822027403	15.045039392042	15.436558266453
H	7.205175382774	16.095646752014	16.609136803561
H	6.326048277191	14.739963849200	15.914794327394
C	6.313050918400	11.750633552172	14.820529255551
H	6.300529536786	12.814668646608	14.571340557775
H	5.341295344290	11.336890213813	14.529849307348
H	7.067141867397	11.275744435762	14.186082781447
C	7.656936887213	7.298843011618	19.379330055963
H	8.203565853919	7.761106261685	20.205089809674
H	6.612692747022	7.200026071248	19.697159049683
H	7.679081997382	7.997177324477	18.541033684431
C	10.735688936023	4.344774247430	18.039396431567

H	10.689588953915	3.871712783215	19.031822112859
C	8.152417994792	4.962348420863	20.207802721891
H	8.483676482679	3.950792851114	19.957025070286
H	7.118240242613	4.886091415072	20.561265211322
H	8.754785928287	5.311543776362	21.052903554804
C	9.857480238291	3.505127052934	17.094061892752
H	8.814542180545	3.459554051831	17.419359568970
H	10.225658609792	2.475019504807	17.034830364843
H	9.867596177204	3.906334290317	16.076314138189
C	9.078416260002	7.011782318698	15.803418712539
H	9.338307637514	6.082479462946	15.287740220270
H	9.071412090126	7.803253775788	15.046448437698
H	8.055324502641	6.908246684834	16.174835102889
C	12.207575706617	4.311871638208	17.593051578181
H	12.325487705858	4.678386868867	16.570413720725
H	12.592956817696	3.286370228896	17.611445020587
H	12.851118126240	4.917356981934	18.236495173386
C	10.083256502493	7.341943999980	16.921264560091
H	9.764389050730	8.284022704920	17.389245277736
C	11.488687848867	7.579447682951	16.341887913118
H	12.213672654074	7.843988913366	17.114925811371
H	11.471005406127	8.398783346950	15.616484470921
H	11.863878639351	6.697147041564	15.817470925059

SS-skew (opt.@T_1)

E(UB3LYP) = -7768.01406771 Hartree

S	0.233408460970	3.228970747934	0.858547240815
C	-0.938798634847	4.315407003772	0.174094035530
C	-1.649856483395	3.675922104848	-0.820249194579
H	-2.430120689721	4.149886014670	-1.401444176601
C	-1.265570665199	2.329883249975	-1.027517073217
C	-0.271571968569	1.898771177948	-0.163958871693
C	0.404977139734	0.609452178220	-0.042906293155
O	1.639979669014	0.564127634404	0.151651828580
Br	-1.987303182450	1.315977504602	-2.459181344400

Si	-1.094905961987	6.118916531472	0.724484693302
C	-0.601063848500	6.185672616382	2.564396259962
H	0.385044602819	5.698074442432	2.586687256582
C	-1.522881738067	5.382527226781	3.498816025542
H	-1.084574839171	5.305855694809	4.499839474939
H	-1.699332487302	4.365459333106	3.140034709915
H	-2.496088503001	5.866719564630	3.612402623387
C	-0.406290388566	7.619592940808	3.089751006135
H	-1.350968315432	8.170487229540	3.116152351546
H	0.294860389967	8.197844379317	2.482100272354
H	-0.015903325586	7.606404261792	4.113234942775
C	0.149230412773	7.141805693103	-0.300084578621
H	0.025822591564	8.182718122000	0.029950249538
C	1.606965735258	6.731574579396	-0.028161812560
H	1.793700346871	5.695450729986	-0.326137912790
H	1.878912949804	6.825367877798	1.026872299502
H	2.300038614871	7.359898813233	-0.598302922986
C	-0.157800420741	7.083782470477	-1.806432149094
H	0.560352421877	7.685594137752	-2.374097002779
H	-1.155823750941	7.462756403582	-2.044017518442
H	-0.092569577741	6.059206236098	-2.186197554386
C	-2.869276698979	6.658781580457	0.283507849334
H	-2.937349584638	6.465115218372	-0.796886122839
C	-3.103161444189	8.167191639644	0.485394151097
H	-4.089678053539	8.459583733035	0.109308238469
H	-2.362292621116	8.780360587588	-0.035003068428
H	-3.072181328032	8.440341821640	1.544194883732
C	-3.979386805818	5.837736618250	0.962972959214
H	-4.041072263833	6.054730237521	2.032057421809
H	-3.828523576719	4.760841259335	0.855250287921
H	-4.956612197309	6.079527087931	0.530563542005
S	-0.233408460970	-3.228970747934	0.858547240815
C	0.938798634847	-4.315407003772	0.174094035530
C	1.649856483395	-3.675922104848	-0.820249194579
H	2.430120689721	-4.149886014670	-1.401444176601
C	1.265570665199	-2.329883249976	-1.027517073217

C	0.271571968569	-1.898771177948	-0.163958871693
C	-0.404977139734	-0.609452178220	-0.042906293155
O	-1.639979669014	-0.564127634404	0.151651828580
Br	1.987303182450	-1.315977504602	-2.459181344400
Si	1.094905961987	-6.118916531472	0.724484693302
C	0.601063848500	-6.185672616382	2.564396259962
H	-0.385044602819	-5.698074442432	2.586687256582
C	1.522881738067	-5.382527226781	3.498816025542
H	1.084574839171	-5.305855694809	4.499839474939
H	1.699332487302	-4.365459333106	3.140034709915
H	2.496088503001	-5.866719564630	3.612402623387
C	0.406290388566	-7.619592940808	3.089751006135
H	1.350968315432	-8.170487229540	3.116152351546
H	-0.294860389967	-8.197844379317	2.482100272354
H	0.015903325586	-7.606404261792	4.113234942775
C	-0.149230412773	-7.141805693103	-0.300084578621
H	-0.025822591564	-8.182718122000	0.029950249538
C	-1.606965735258	-6.731574579396	-0.028161812560
H	-1.793700346871	-5.695450729986	-0.326137912790
H	-1.878912949804	-6.825367877798	1.026872299502
H	-2.300038614871	-7.359898813233	-0.598302922986
C	0.157800420741	-7.083782470477	-1.806432149094
H	-0.560352421877	-7.685594137752	-2.374097002779
H	1.155823750941	-7.462756403582	-2.044017518442
H	0.092569577741	-6.059206236098	-2.186197554386
C	2.869276698979	-6.658781580457	0.283507849334
H	2.937349584638	-6.465115218372	-0.796886122839
C	3.103161444189	-8.167191639644	0.485394151097
H	4.089678053539	-8.459583733035	0.109308238469
H	2.362292621116	-8.780360587588	-0.035003068428
H	3.072181328032	-8.440341821640	1.544194883732
C	3.979386805818	-5.837736618250	0.962972959214
H	4.041072263833	-6.054730237521	2.032057421809
H	3.828523576719	-4.760841259335	0.855250287921
H	4.956612197309	-6.079527087931	0.530563542005

SO-planar (1) (opt.@S_0)

E(B3LYP) = -7445.10860935 Hartree

C	3.804961309869	-0.787018542057	0.601389864492
C	3.980387461210	-2.134714523444	0.804568170279
H	4.850264046999	-2.719566659025	0.549118387202
C	2.795659479623	-2.615767616758	1.420622518495
C	1.951705414065	-1.537731836994	1.577386419881
C	0.611691974725	-1.381953684638	2.117530391902
O	-0.089485249961	-2.317083224246	2.461109302714
Br	2.475948314545	-4.396703804604	1.927780749468
Si	4.857851776600	0.628151572721	-0.087032770462
C	3.754405691651	1.558998125523	-1.328879329124
H	2.987604604972	2.021105789701	-0.692930515477
C	3.016745468284	0.656106740410	-2.332927030056
H	2.317212678128	1.245719184935	-2.936045418177
H	2.437849456440	-0.124576508147	-1.832728576807
H	3.703865226965	0.164849406441	-3.026330875378
C	4.507416795927	2.692211048270	-2.048789273539
H	5.265118221771	2.298782927532	-2.733576032174
H	5.011458760675	3.368744430234	-1.351894134697
H	3.818631406185	3.297714226660	-2.647988225232
C	5.293979753741	1.751475321974	1.388360031257
H	5.968238471493	2.524502443737	0.992805556747
C	4.062365046362	2.457877016940	1.986968498980
H	3.312672539446	1.749630295453	2.348768092365
H	3.568764957822	3.112970717927	1.264096756247
H	4.357208944304	3.082491168894	2.837529750447
C	6.055425821169	0.972643457436	2.476934420691
H	6.349244697592	1.637252624213	3.296430871268
H	6.967017267626	0.500529368617	2.098453739021
H	5.430857111443	0.184749875379	2.909360796801
C	6.428445738172	-0.190833161509	-0.789221058920
H	6.742272437140	-0.889163434655	0.000433307972
C	7.585235384587	0.803213766376	-1.002342580588
H	8.488830536404	0.279228531799	-1.332928134218

H	7.840872867925	1.347571012128	-0.089789753655
H	7.345589410266	1.544141385976	-1.770574909309
C	6.181646229580	-1.016170426668	-2.064888127890
H	5.951381789942	-0.370092822917	-2.916691730147
H	5.353817336047	-1.722348577496	-1.956369351629
H	7.073992317045	-1.592507020349	-2.333182552722
C	-3.483471835421	0.381158311388	0.682152816040
C	-3.059455661009	1.642595942632	1.043467545811
H	-3.632097094019	2.545256516126	0.874981003423
C	-1.790616282637	1.656075344845	1.668293107481
C	-1.210784716604	0.404383578159	1.794640375764
C	0.119730769628	0.070005121005	2.313008191326
O	0.835206457207	0.849875490965	2.909516007120
Br	-1.012760939737	3.298068896310	2.216949265762
Si	-5.078541999264	-0.082461828633	-0.228460580703
C	-6.339472635584	1.291358436083	0.166987298636
H	-5.835957415883	2.209248741805	-0.169657242920
C	-6.650425585931	1.467475414988	1.663702214711
H	-7.237774678233	2.377217444313	1.830502400597
H	-5.745806136991	1.543040143838	2.272064758779
H	-7.237924901191	0.632340482440	2.052333682356
C	-7.637855730692	1.164143573288	-0.650661909325
H	-8.212754895766	0.281420544021	-0.356022535322
H	-7.451596660851	1.092456421033	-1.725827128497
H	-8.283905645434	2.034065713917	-0.489462733534
C	-4.688049149964	-0.061919946403	-2.096825772033
H	-5.628258286403	-0.320561280227	-2.603381795545
C	-4.257596055811	1.332624358048	-2.583486613815
H	-3.345366386143	1.666087468668	-2.078373515561
H	-5.027216313197	2.090525181615	-2.412355201519
H	-4.046932876683	1.322648127339	-3.658558415055
C	-3.638920700725	-1.118239661521	-2.485329198653
H	-3.463599812873	-1.112535053911	-3.566755633434
H	-3.943759472672	-2.132214299277	-2.212222762426
H	-2.676737439132	-0.922641445723	-2.002365188033
C	-5.532611849424	-1.855460875007	0.302141607599

H	-4.611832853580	-2.428046498172	0.115973774115
C	-6.637309829245	-2.484181821615	-0.566750012752
H	-6.786055529820	-3.536111708120	-0.299837203269
H	-6.402988149415	-2.449991773393	-1.634078165261
H	-7.597769237003	-1.980360963977	-0.424298521744
C	-5.863044042873	-2.006705058537	1.797521855384
H	-6.824308537362	-1.547630224195	2.042283925429
H	-5.105493316076	-1.554255750626	2.442181668641
H	-5.934187344133	-3.065016525661	2.070615017138
O	2.572139663201	-0.429332068921	1.068648343846
S	-2.298318389037	-0.799182064671	1.127852023415

SO-skew (3) (opt.@S_0)

E(B3LYP) = -7445.11383761 Hartree

Br	9.733020582529	5.852611410061	5.259673298106
Br	8.232927819445	6.980082703480	0.781607154257
S	7.191702047070	7.835659650347	8.304638457103
Si	4.739025770390	3.729549358653	4.274103269779
Si	8.350128330933	6.373765013680	10.889971233461
O	6.200985676713	6.094424449140	4.180839133218
O	6.203971432826	9.000167098280	5.770018304220
O	8.370338636211	8.835317124692	3.631884225211
C	7.021817894879	6.899653643878	3.435564859578
C	7.122536506362	8.224206434925	5.586038704884
C	8.783502105665	6.567982388271	6.744451035381
C	7.183947325923	6.328326475795	2.196960175566
C	7.775848410224	7.512934265817	6.686960854560
C	6.433589666983	5.121652174227	2.190555529952
H	6.349980921584	4.426800535376	1.369399193685
C	9.082423517135	6.125562423873	8.055980497336
H	9.853270267780	5.393866946717	8.260303679737
C	7.605609655229	8.036915845665	4.128026048649
C	7.272451215912	4.829946295261	11.198759957986
H	7.273706281231	4.671107626860	12.285878946638

C	8.305965505880	6.721434190416	9.026563853704
C	4.509934405354	2.328693526126	3.001482373392
H	5.518452968893	2.167896379984	2.593601320746
C	5.844361880653	5.008598796150	3.425699778435
C	10.165669122021	5.977585766347	11.313578140297
H	10.386253319322	5.088295358400	10.705369427112
C	5.691099478017	3.087977730750	5.795722713035
H	5.101879321072	2.244343143256	6.181686897584
C	7.855735083506	3.567956641775	10.540407614940
H	7.960112567291	3.691569293416	9.458195717759
H	7.201360582107	2.704583756689	10.704182685083
H	8.839535029838	3.308121310053	10.940609420953
C	7.572018749938	7.875622300440	11.768084985941
H	6.632413225354	8.047043864495	11.222205002348
C	4.059604230596	1.000058811744	3.636970483339
H	4.750265890343	0.652042800791	4.409164959964
H	3.996300838553	0.211949164621	2.878587753323
H	3.069424157359	1.084413426712	4.094491229380
C	3.588711981192	2.702438140318	1.825742239471
H	2.547533121813	2.785278762585	2.149588292426
H	3.621135261440	1.931728424092	1.047802266773
H	3.861235269635	3.652829033631	1.358955088651
C	5.815894383812	5.057284220661	10.756017442076
H	5.352640903987	5.906224276776	11.266595524583
H	5.201456635503	4.175633514719	10.969455920663
H	5.749199082995	5.244096017380	9.680837926109
C	10.352806314721	5.575626380307	12.788052824823
H	10.185417749283	6.424151197472	13.457887318236
H	11.374555424473	5.224079584516	12.968030994593
H	9.674280051663	4.774371882247	13.094907431742
C	7.193716232566	7.592757155505	13.233910633863
H	6.533354620489	6.727939409478	13.337380430947
H	6.673387866065	8.452670800147	13.669393837558
H	8.077253554117	7.407923010526	13.851751017245
C	5.808550153767	4.128154124768	6.923866525218
H	6.341964553301	5.024119943186	6.597453267140

H	6.363563381644	3.711418587577	7.769774328484
H	4.833518596531	4.443671458112	7.303743095161
C	3.161875060985	4.643230642539	4.824362947366
H	3.519590940504	5.323336126345	5.609247577797
C	11.176904057834	7.064025708421	10.907466361291
H	11.064092008123	7.366997227061	9.863701115304
H	12.202725245377	6.701661848888	11.037045141194
H	11.074599470295	7.961263444464	11.522087984044
C	7.082483481460	2.555501840436	5.406628364557
H	7.035883526720	1.782288779127	4.633898201162
H	7.588293087908	2.118006243324	6.274539397001
H	7.723151329251	3.359260874061	5.032103202818
C	8.402120067719	9.167380827588	11.661791615397
H	9.305142803353	9.110768522874	12.275456460698
H	7.825162265741	10.026654609004	12.020008445817
H	8.711118921361	9.386664522868	10.636725736407
C	2.517740758964	5.520617077871	3.736949066491
H	3.230979241143	6.227835158665	3.305870841846
H	1.692098363664	6.105582014833	4.156784534219
H	2.106567668833	4.924942101196	2.918339081671
C	2.129874795465	3.696075247647	5.462400671116
H	1.704358450419	3.010369015385	4.723122818531
H	1.295438820014	4.261482644734	5.891306544159
H	2.558454074749	3.089641471502	6.266398568707

SO-(4) (opt.@S_0)

E(B3LYP) = -7445.10794075 Hartree

C	-3.510436725169	-0.463266250380	0.060058361848
C	-3.927126131716	0.665104258676	0.720894054164
C	-1.809527317047	0.325555738225	1.310712534614
C	-2.158742632552	-0.680020269961	0.436261279616
C	-0.572087316021	0.748203352321	1.951960404012
O	-0.451714686624	1.796282198628	2.552715104458
C	0.615928805059	-0.230325654416	1.960619804831
O	0.628291966653	-1.069770333722	2.841808711923

C	1.718049643571	-0.060786181455	1.015883343245
C	1.863742683350	0.690964019858	-0.134019410207
C	3.104427804344	0.502233316571	-0.789667710879
C	3.929204237714	-0.400271187939	-0.153113186290
S	3.153671755942	-1.026953621146	1.266041912402
Br	0.557327894199	1.859675556455	-0.873956476396
H	-4.089724672955	-1.068148983620	-0.619891585252
H	3.365601574460	1.024461364751	-1.700821939712
O	-2.891590743711	1.140813445831	1.478697020042
Br	-1.069192536768	-2.069755650298	-0.223580302659
Si	-5.517950732927	1.689964330454	0.743063164950
Si	5.630944894306	-1.001298623279	-0.730206351938
C	-6.744819700898	0.758564806056	-0.379780625688
H	-6.152019405217	0.498662690580	-1.268904085305
C	-6.022910029527	1.892418429521	2.566501289431
H	-5.236097712858	2.536253380341	2.981732682117
C	-5.076668456474	3.379947659291	-0.021021608345
H	-6.021858456973	3.935012126196	-0.101830163608
C	-7.915582633184	1.638626003457	-0.855949371097
H	-8.555510622549	1.085840127384	-1.552559131418
H	-8.548832725652	1.955774825834	-0.022326706683
H	-7.576709047035	2.540296565622	-1.372348415211
C	-7.269034693433	-0.555555999858	0.227222426323
H	-6.466600733764	-1.200877056216	0.595026498091
H	-7.943765920967	-0.363397254729	1.066105391273
H	-7.837327767192	-1.126360836379	-0.515512442051
C	-6.006283184807	0.586944946892	3.381047058216
H	-5.042613428263	0.075542071631	3.315162709773
H	-6.193496155546	0.794539092192	4.440363628159
H	-6.776515235488	-0.113959197070	3.049699130451
C	-7.363859404911	2.631515364057	2.726213707213
H	-8.201335542328	2.024610163184	2.368307817820
H	-7.560242873673	2.856787755111	3.780055209905
H	-7.387631005515	3.580617307783	2.181832030767
C	-4.120093525613	4.203770305079	0.861499041573
H	-3.183083139144	3.673934291516	1.053442732331

H	-3.867558671070	5.151597519952	0.373209188576
H	-4.561056129494	4.448614085838	1.831296985729
C	-4.501296223635	3.217064005062	-1.440123549759
H	-4.303745829959	4.193863008126	-1.894822921336
H	-3.552911815576	2.670623276753	-1.422018997967
H	-5.177761544280	2.677648296318	-2.109701666655
C	6.599955998636	-1.522026314798	0.825464335092
H	5.916214937586	-2.213249147152	1.340336616025
C	6.367788512615	0.410076685269	-1.778336697651
H	5.620403274647	0.561861235887	-2.570657144585
C	5.342554169579	-2.528372612772	-1.838452473979
H	6.339360581303	-2.852989295422	-2.168053561463
C	4.698755464801	-3.694531069927	-1.068129767898
H	3.701308715164	-3.430896598710	-0.703571721840
H	4.584125336039	-4.571136338527	-1.715109140080
H	5.292646039177	-4.005553571880	-0.204275392254
C	4.518213395802	-2.188169966230	-3.092051122443
H	3.524416742182	-1.814569806175	-2.825068918188
H	5.000766469196	-1.430999467173	-3.716439156490
H	4.372721060421	-3.076899728560	-3.715872168308
C	7.684088774486	0.012918757322	-2.471341494148
H	7.591861951531	-0.909991631855	-3.050649161761
H	8.011336451194	0.798807677617	-3.160803849472
H	8.490919293392	-0.131535230937	-1.746985366797
C	6.522050017117	1.749941885381	-1.037424996965
H	5.606485073840	2.049866009557	-0.521806962142
H	7.319016820323	1.706833384806	-0.291383289339
H	6.783318040370	2.549837804958	-1.739082481532
C	7.882100538300	-2.312169245820	0.505435631914
H	8.625417190429	-1.687497186064	0.001645885504
H	8.346922855962	-2.682278714466	1.425685589520
H	7.692882506335	-3.178526639170	-0.133923835486
C	6.898455398884	-0.370649512388	1.801931803285
H	6.011859063273	0.225825228762	2.031130687361
H	7.284669796210	-0.760166773649	2.749999710967
H	7.658063482121	0.305444337036	1.401169197752
