# Supplementary material for: Photoinduced crystal melting with luminescence evolution based on conformational isomerisation
Source: Chem Sci. 2023 Apr 20;14(20):5302–8. doi: 10.1039/d3sc00838j (PMC10207888; doi:10.1039/d3sc00838j)
Supplement: SC-014-D3SC00838J-s004 [file SC-014-D3SC00838J-s004.pdf]

Electronic Supplementary Material (ESI) for Chemical Science.
This journal is © The Royal Society of Chemistry 2023

Electronic Supplementary Information

for

Photoinduced Crystal Melting with Luminescence Evolution Based on Conformational Isomerization

Mao Komura, Hikaru Sotome, Hiroshi Miyasaka, Takuji Ogawa and Yosuke Tani*

Department of Chemistry, Graduate School of Science, Osaka University
1-1 Machikaneyama, Toyonaka, Osaka 560-0043, Japan
E-mail: y-tani@chem.sci.osaka-u.ac.jp

Table of Contents

1. Instrumentation and chemicals 2
2. Procedures for evaluating photoinduced crystal melting 4
3. Evaluating photoresponse before macroscopic melting 9
4. Theoretical study on the conformational dynamics of **SO** 16
5. Photoresponses and structures of analogous diketones 23
6. References 32

1. Instrumentation and chemicals

All the diketone derivatives (**SO**[1a], **OO**,[1a] and **SS** [1c]) were synthesized and confirmed to be highly pure using previously reported methods. The crystal samples were prepared through recrystallization by cooling a MeOH solution. UV-vis absorption and photoluminescence (PL) spectra were recorded at room temperature (RT) in air unless noted otherwise. The UV-vis absorption spectra were acquired using a Shimadzu UV-3150 spectrometer. PL spectra of the crystals and the supercooled liquid (SCL) were acquired using a JASCO FP-8200 spectrofluorometer. PL spectral tracking during the photo-induced crystal-to-liquid transition (PCLT) was performed using instrumentation A or B: (A) an OLYMPUS BX60M fluorescence microscope equipped with a Ushio 100 W ultrahigh-pressure mercury lamp (USH-102D), OLYMPUS mirror units (U-MWUS, λ_{max} = 365 nm, 60 mW cm^{-2}), and a HAMAMATSU Photonics PMA-12 photonic multichannel analyzer (C14631-01) and (B) a laboratory-built hyperspectral imaging system, which is described below (see Figure S1). Photographic images and movies were recorded using a SONY NEX-5N camera attached to the OLYMPUS BX60M fluorescence microscope with a MICRONET NY-1S adaptor unless noted otherwise. An OLYMPUS mirror unit (U-MBFL3, longpass, λ > 400 nm, < 10 mW cm^{-2}) was used for recording the bright-field and polarizing images while a U-MWUS or U-MWBS (λ = 460 to 490 nm) unit was used for the PL images.

Details of the laboratory-built hyperspectral imaging system (B)

The illumination light source was an ultraviolet light emitting device (LED, Thorlabs, M365L2) centered at 365 nm, and its output was used for initiation of PCLT and photoexcitation of the sample to induce the emission. The **SO** crystal on the cover slip was placed on a 3-axis XYZ translation stage (OptoSigma, TSD-1202SH-M6 and Thorlabs, NFL5DP20/M) of a home-built microscope. The output of a white LED (Thorlabs, MCWHL6), which is combined to the UV light with a dichroic mirror (Thorlabs, DMLP425R), was used for bright-filed imaging. The emission from the sample and transmitted/scattered UV light were collected with an objective lens (Olympus, PLN10X) and split into two portions using a beam splitter with a ratio of 1:1. One was focused onto a complementary metal oxide semiconductor (CMOS) color camera (Mightex, SCE-C030-U) with an achromatic lens (Thorlabs, AC254-100-A-ML, f = 100) for recording bright-filed images. The other part was filtered by a long-pass filter (Semrock, BLP01-405R-25) to remove the excitation light. The extracted emission was imaged onto the adjustable slit

(Thorlabs, VA100/M) with an achromatic lens (Thorlabs, AC254-100-A-ML, f = 100) for selection of the observation area of interest for the spectral detection. The spatially selected emission was spectrally dispersed by a transmission grating (RSpec, SA-100, 100 groove/mm), which is usually used in the field of astronomical spectroscopy, and the 0th and 1st order diffracted signals were recorded with a CMOS camera (FLIR, GS3-U3-23S6M-C) as one-line emission image and the corresponding spectra. The observation wavelength was calibrated by measuring transmission spectra of a V10 filter (HOYA, V10) which shows narrow absorption peaks as a source of the white LED. The emission spectral images were acquired at 5 frames per second. The bright-field images were recorded before and after the spectral imaging so as to confirm changes in the color and morphology of the crystalline sample. The overall setup was controlled by a home-built program written in LabVIEW 2018.

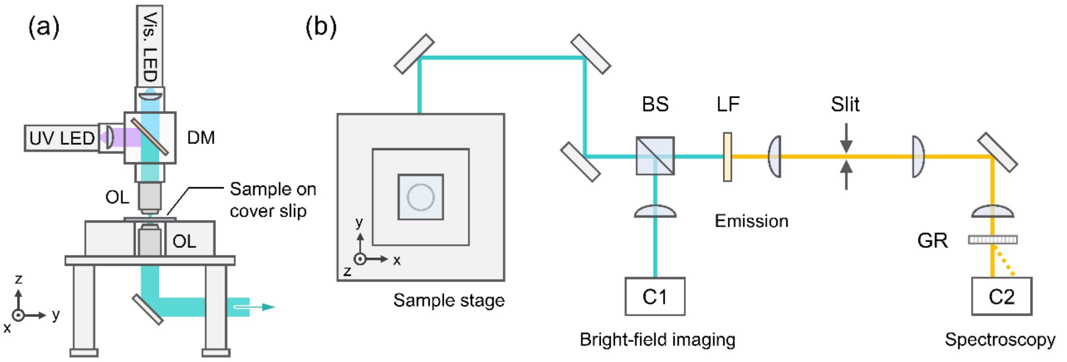

Figure S1. Schematic diagram of a hyperspectral imaging system used in the present work. (a) Side view. (b) Top view. DM: dichroic mirror, OL: objective lens, BS: non-polarizing beam splitter, LF: longpass filter, Slit: adjustable slit, GR: transmission grating, C1: CMOS camera for bright-field imaging, C2: CMOS camera for spectroscopy.

Details of a home-built Raman microscope

The light source was a He-Ne laser (Thorlabs, HNL210LB) centered at 632.8 nm. The output was spectrally cleaned up by a volume Bragg bandpass filter (OptiGrate, BP-633-M) and the spot was magnified by a factor of 3 with a beam expander for matching with pupil of an objective lens. The laser light was introduced into an inverted microscope (Olympus, IX73) and focused to the sample on the cover slip by the objective lens (Olympus, LUCPLFLN60X, NA 0.7). Raman scattering light was collected with the same objective lens and guided into a spectrograph with a half mirror (50:50). Unwanted Rayleigh scattering was effectively removed by three volume Bragg notch filters (OptiGrate, BNF-633-OD3-11M). Raman spectra were detected by a charge-coupled device (Andor, DU401A-BFV-SG) equipped with a polychromator (Jobin Yvon, HR-320, f = 320 mm,

1200 grooves/mm). The wavenumber calibration was performed using line emission of a neon lamp. The wavenumber resolution was ca. 4 cm^{-1}. Ultraviolet light at 365 nm (Thorlabs, M365L2) was irradiated to the sample through an optical fiber for initiation of PCLT. The Raman spectra were recorded at every 1 second during the PCLT process and 10 spectra were typically averaged into 1 spectrum. The bright-field images were also monitored by a monochrome CMOS camera (FLIR, GS3-U3-23S6M-C) for tracking the morphological change of the sample. The overall setup was controlled by a home-built program written in LabVIEW 2018.

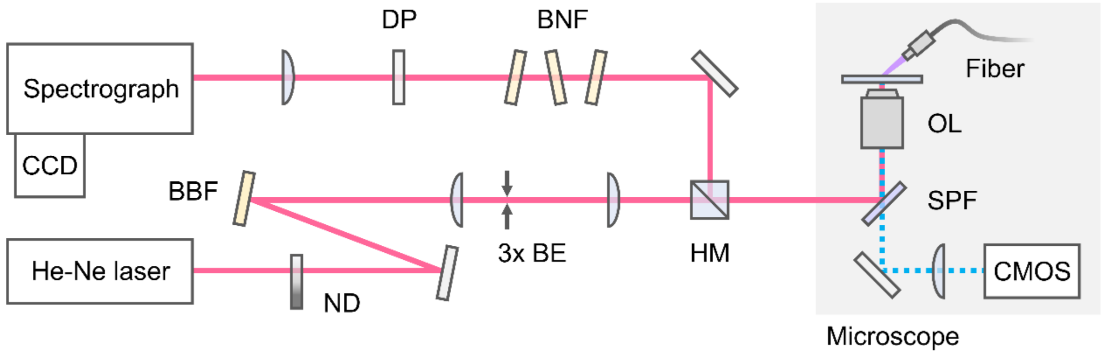

Figure S2. Schematic diagram of a Raman microscope used in the present work. ND: neutral density filter, BBF: volume Bragg bandpass filter, BE: beam expander (3x), HM: half mirror (50:50), SPF: shortpass filter (cutoff 505 nm), OL: objective lens (60x, NA 0.7), BNF: volume Bragg notch filter, DP: depolarizer, CCD: charge-coupled device, CMOS: complementary metal oxide semiconductor camera.

2. Procedures for evaluating photoinduced crystal melting

PL spectrum of SO crystal during PCLT (Figure 2 and Movies S1, S2)

The **SO** crystal, obtained by recrystallization from MeOH, was placed on a slide glass under a microscope (instrumentation A). Next, UV light was irradiated on the sample through the microscope. Movie was recorded under continuous UV irradiation using an Olympus U-MWUS mirror unit (excitation, 330–385 nm; emission, >420 nm). Photographic images of Figure 2a were captured from the movie, while Movies S1 and S2 were obtained by changing the speed. Fluorescence microscope images in Figure 2b were taken through an Olympus U-MWBS mirror unit (excitation, 460–490 nm; emission, >500 nm).

Luminescence lifetime of SO crystal after UV-irradiation.

The **SO** crystal, obtained by recrystallization from MeOH, was placed on a slide glass under a microscope (instrumentation A). Next, UV light was irradiated on the sample through the microscope for 1 minute. The decay curve of the PL intensity at 575 nm was acquired with a HORIBA DeltaFlex multichannel scaling system using DeltaDiode for excitation (368 nm). The area-weighted average lifetime τ_p = 15 μs was obtained from a triple-exponential fit using a HORIBA EZTime software (τ_1 = 16 μs (55%), τ_2 = 5.0 μs (12%), τ_3 = 40 μs (33%)). The reduced chi-square of the curve fitting was 1.10.

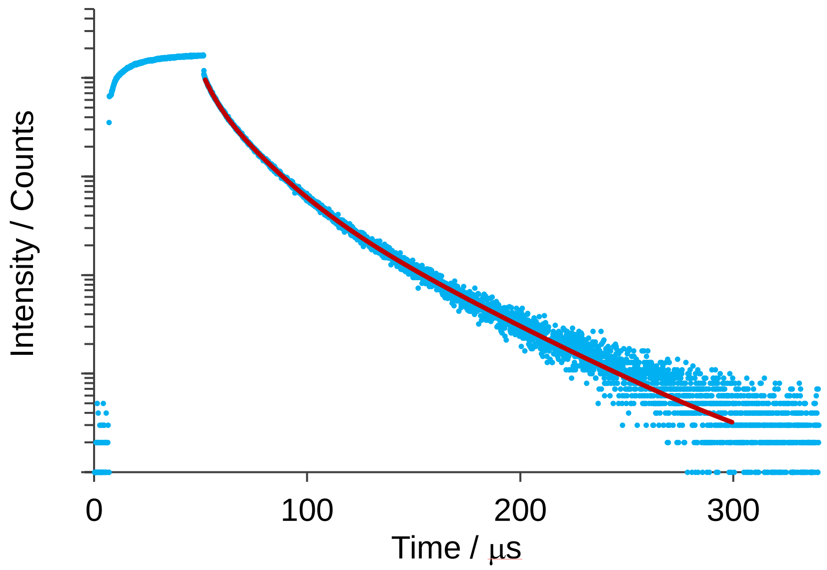

Figure S3. Photoluminescence decay curve of **SO** crystal after UV irradiation for 1 min at RT in air. The red line is the fitted curve. PL intensity was recorded at 575 nm (λ_{ex} = 368 nm).

PL spectra of pristine SO powder during PCLT

The pristine **SO** powder, obtained by the spontaneous crystallization of the SCL, was placed on a slide glass under a microscope (instrumentation A). Next, UV light was irradiated on the sample through the microscope, and the PL spectra were acquired after <1, 10, and 30 s irradiation (Figure S4). The PL spectrum of the SCL, obtained as previously reported,[1a] is shown for comparison. The PL spectra almost perfectly overlapped that of **SO** in the SCL state. Based on the results of our previous study,[1a] we concluded that the emerging emission is of the planar conformer.

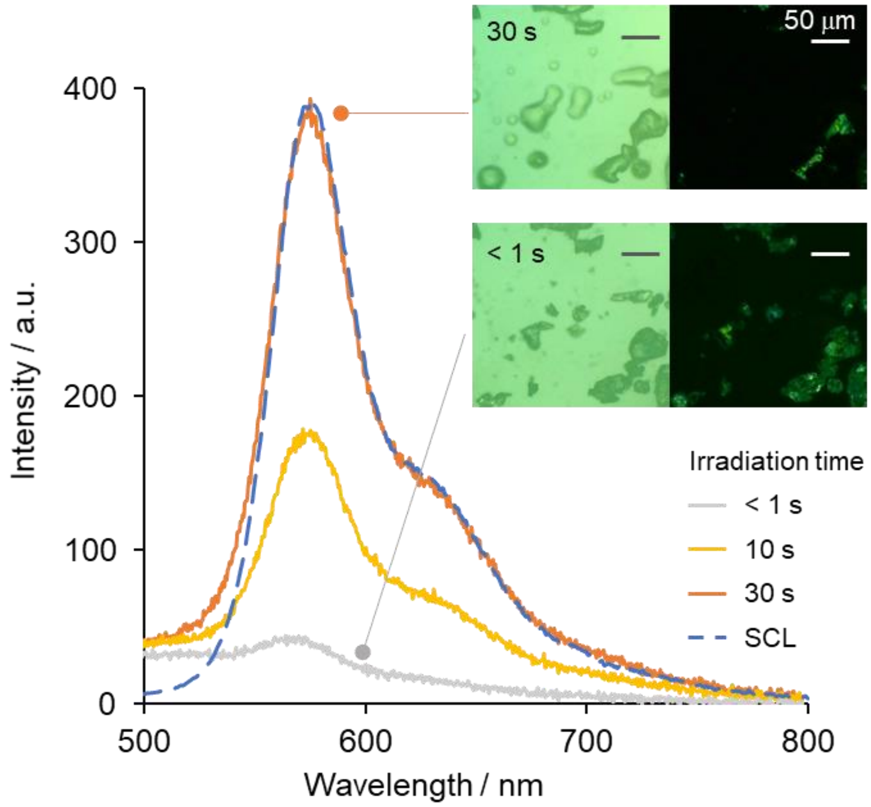

Figure S4 Photoluminescence spectra of **SO** pristine powder and SCL. Inset: photographic images under bright-field (left) and polarizing (right) optical microscope before and after irradiation.

PL spectra of SO SCL at higher temperatures

The **SO** SCL was placed in a quartz tube in an Ar atmosphere, and the tube was capped with a rubber septum. The PL spectra of the SCL were acquired using a JASCO FP-8200 spectrofluorometer equipped with a CoolSpeK USP-203-B cryostat (UNISOKU) with an L42 sharp cut filter (HOYA, longpass, > 420 nm) and a U340 bandpass filter (HOYA, λ_{ex} = 368 nm). The emission was mostly quenched at 55 °C, which is lower than the melting point (59.5 °C), even in the Ar atmosphere. Therefore, the increase in the emission intensity in air during melting by continuous UV irradiation (Figure S5 and Figure 3) indicated that the temperature was not high enough to cause thermal melting.

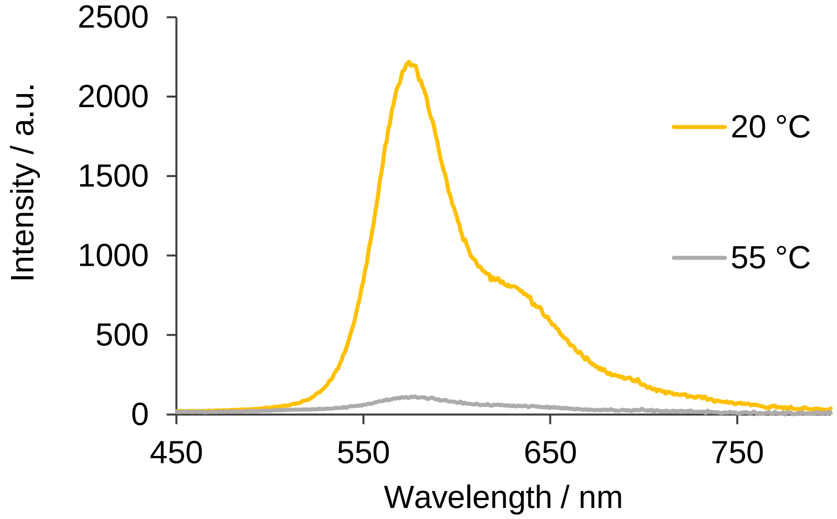

Figure S5. Steady-state PL spectra of **SO** SCL at 20 and 55 °C.

Excitation-wavelength-dependence of photoresponse

The **SO** crystal was placed on a slide glass under a microscope (instrumentation A). After obtaining the bright-field and polarizing photographic images of the crystal with a mirror unit (U-MBFL3), the mirror unit was switched to U-MWBS to irradiate the sample with blue light (BL) (Figure S6b and Figure S7a–b). Photographic images of the samples were recorded, and the mirror unit was switched to U-MWUS to irradiate the sample with UV light for the indicated periods.

Irradiation with BL resulted in no discernible changes in the bright-field and polarizing images even after more than 30 min (Figure S6a and 7b). In contrast, some of the crystal particles exhibited noticeable melting (i.e., their birefringence was faded, and their outlines became rounder) under UV irradiation for 2.5 min (Figure S7c). The UV-vis absorption spectrum of the **SO** crystal indicated that the crystal absorbs much light in the UV region than in the BL region. Therefore, the melting of crystal is triggered by light absorption.

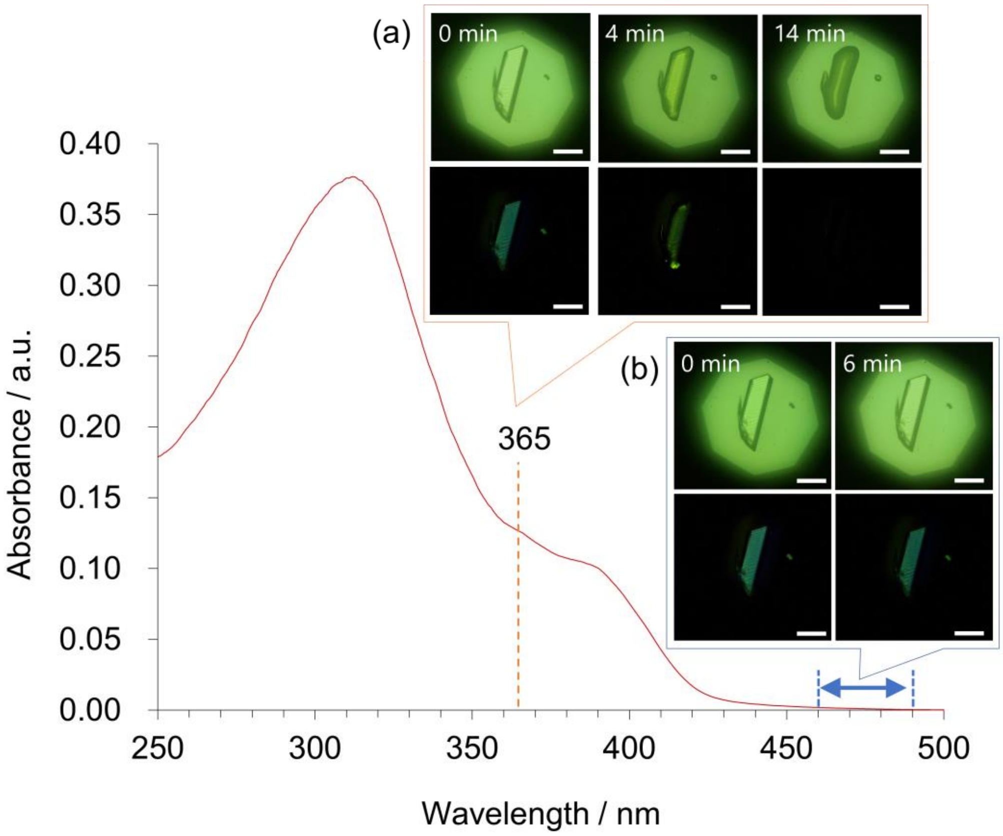

Figure S6. Absorption spectrum and photographic images of **SO** crystal obtained using (inset, top) bright-field and (inset, bottom) polarizing optical microscopes with crossed Nichol prisms before and after (a) UV-light irradiation and (b) blue-light irradiation for indicated periods. Double-headed arrow represents irradiation wavelength range of blue light. Scale bar: 100 μm.

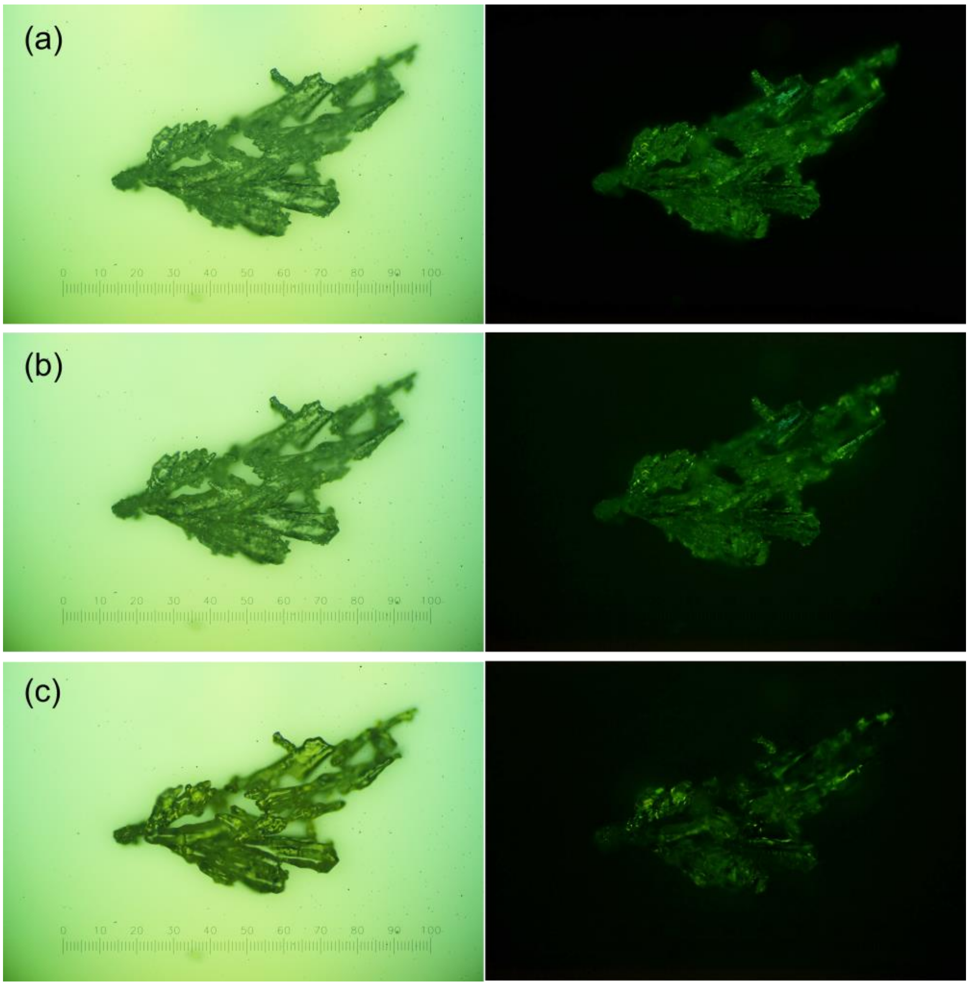

Figure S7. Photographic images of **SO** crystals under (left) bright-field and (right) polarizing optical microscope with crossed Nichol prisms (a) before irradiation, (b) after continuous blue-light irradiation for 32 min, and (c) after continuous UV-light irradiation for 2.5 min. Scale bar: 1.0 mm.

3. Evaluating photoresponse before macroscopic melting

PL spectral tracking during irradiation for short periods (Figures 3 and S8)

A **SO** crystal was placed on a cover strip on the sample stage of instrumentation B, and subjected to the experimental process described in Section 1 (18 mW cm^{-2}). Figures 3 and S8 show two-dimensional representations of the results obtained using a different crystal. The intensity of the transient emission at approximately 520 nm and the length of the inductive period depended on the samples, which suggests that these parameters are determined by the crystallinity and/or thickness of the crystal.

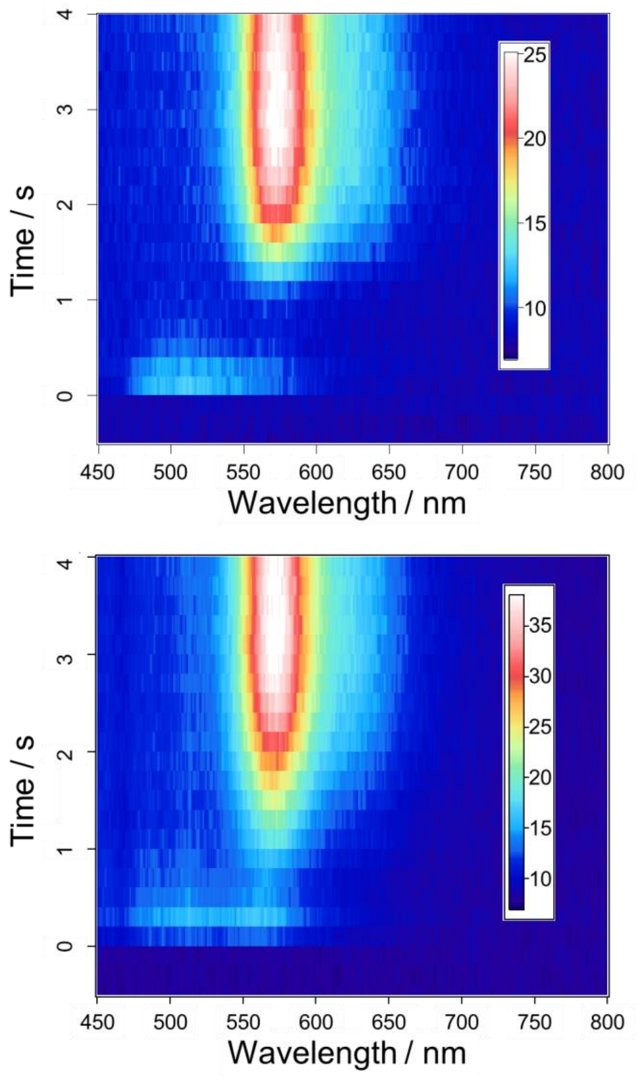

Figure S8. Evolution of PL spectra of different crystal samples during continuous UV-light irradiation. Note that top panel is the same as that in Fig 3a.

Reversibility of the photoresponse

A **SO** crystal was placed on a cover strip on the sample stage of instrumentation A. The PL spectrum was recorded initially (Figure S9, red trace) and after UV irradiation for 4 seconds (yellow trace), confirming the decrease in the intensity and the progress of crystal loosening (1st step). The decrease in the intensity at 515 nm is approximately 30%, which corresponds to only 10% progress for disappearing green emission according to Fig. 3b. Then, the UV irradiation was stopped, and the solid was kept in the dark for 20 minutes. After that, the PL spectrum was recorded (blue trace), which was revealed to be unchanged.

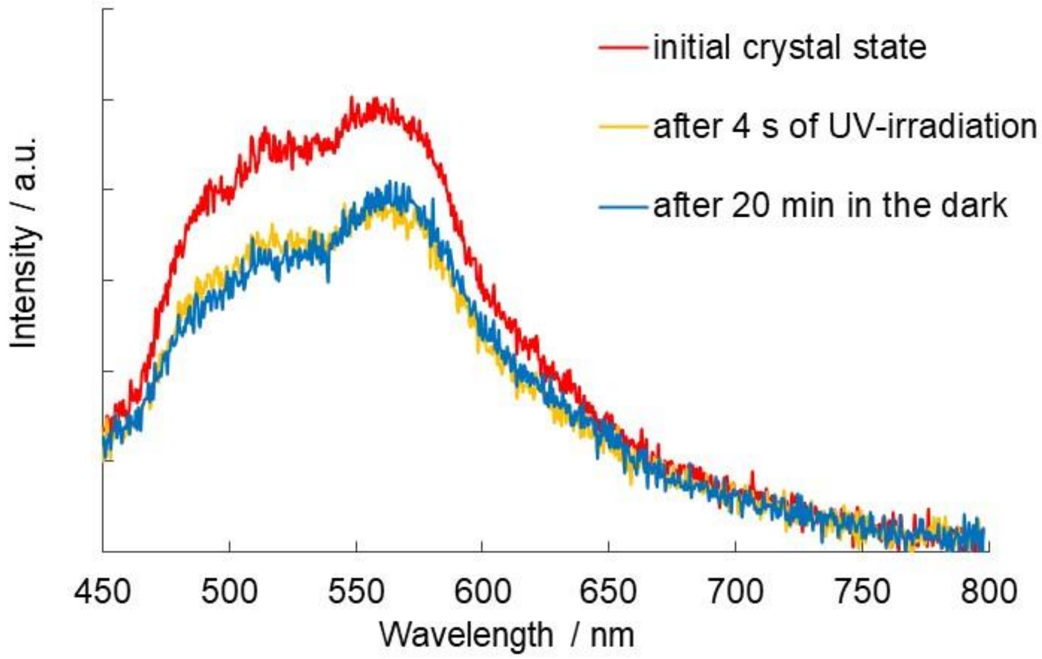

Figure S9. Photoluminescence spectra of **SO** under fluorescence microscope.

Low-temperature PL measurements of SO crystal

The **SO** crystal or SCL was placed in a quartz tube and capped with a rubber septum in air. The PL spectra were acquired at –30 °C using a JASCO FP-8200 spectrometer equipped with a CoolSpeK USP-203-B cryostat (UNISOKU) with an L42 sharp-cut filter (HOYA, longpass, > 420 nm) and a U340 bandpass filter (HOYA) (λ_{ex} = 368 nm). Since the emission of the SCL is from the planar conformer, it can be concluded that the difference in the PL spectra is because of the emission from the skew conformer in the crystal (Figure S10).

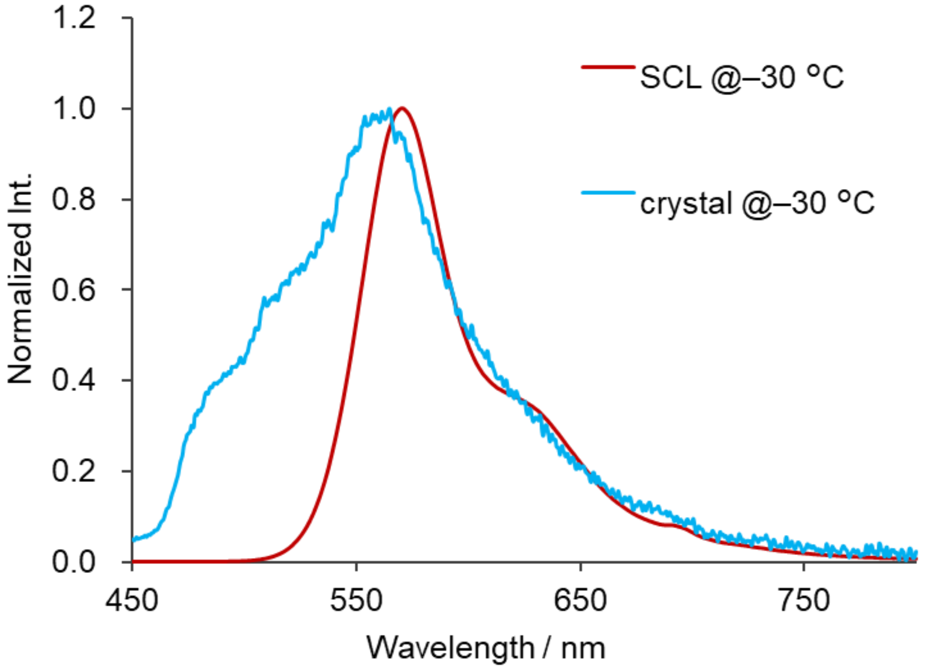

Figure S10. Normalized steady-state PL spectra of **SO** crystal and SCL in air at –30 °C.

Single-crystal X-ray structure analysis (Figure 4)

Single-crystal of **SO** suitable for the X-ray crystal structure analysis was obtained by cooling MeOH solution. Data were successively collected at 123, 223, and 300 K using the same crystal on a Rigaku XtaLAB Synergy-R diffractometer with graphite monochromated MoKα radiation (λ = 0.71075 Å) in the ω-scan mode. The crystal was cooled by a stream of cold N_2 gas. Collection, indexing, peak integration, cell refinement, and scaling of the diffraction data were performed using the CrysAlisPro (Rigaku) software. The structure was solved by direct method (SHELXT 2018) and refined by full-matrix least-square refinement on F^2 (SHELXL2018). The non-hydrogen atoms were refined anisotropically. All hydrogen atoms were placed on the calculated positions and refined using the riding model.

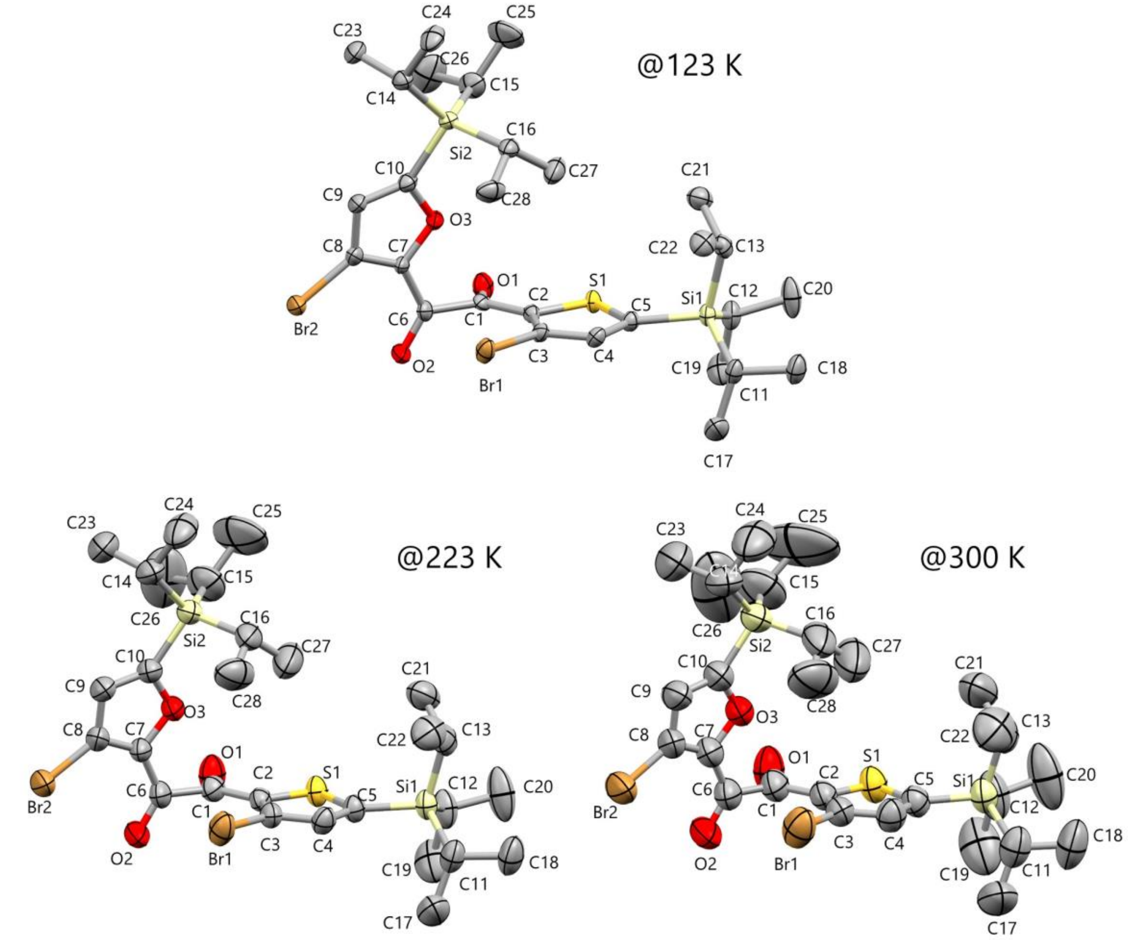

Figure S11. ORTEP drawing of the single-crystal X-ray structures of **SO** at various temperatures. Hydrogen atoms are omitted for clarity, and the thermal ellipsoids are set at the 50% probability level.

Table S1. Crystallographic Data for **SO** (CCDC 2095928, 2189552, 2189553)

Empirical formula	$C_{28}H_{44}Br_2O_3SSi_2$	$C_{28}H_{44}Br_2O_3SSi_2$	$C_{28}H_{44}Br_2O_3SSi_2$
Formula weight	676.69	676.69	676.69
Temperature / K	123	223	300
Crystal system	Monoclinic	Monoclinic	Monoclinic
Space group	$P\ 2_1/n$	$P\ 2_1/n$	$P\ 2_1/n$
a / Å	14.1498(4)	14.1870(4)	14.2291(4)
b / Å	9.2174(3)	9.2501(3)	9.2419(3)
c / Å	25.7482(8)	26.1208(7)	26.5165(7)
α / °	90	90	90
β / °	95.165(3)	95.646(2)	96.382(3)
γ / °	90	90	90
Volume / Å^3	3344.56(18)	3411.23(17)	3465.41(18)
Z	4	4	4
ρ (calcd) / g·cm^{-3}	1.344	1.318	1.297
Crystal size / mm^3	0.4 × 0.2 × 0.1	0.4 × 0.2 × 0.1	0.4 × 0.2 × 0.1
Completeness	99.99%	99.97%	99.97%
Goodness-of-fit on F^2	1.02	1.009	1.009
Final R indexes [I > =2σ(I)]	R_1 = 0.0569, wR_2 = 0.1030	R_1 = 0.0642, wR_2 = 0.1205	R_1 = 0.0627, wR_2 = 0.1736
Final R indexes [all data]	R_1 =0.0958, wR_2 = 0.1135	R_1 = 0.1273, wR_2 = 0.1394	R_1 = 0.1517, wR_2 = 0.2132
CCDC number	2095928	2189552	2189553

Single-crystal X-ray structure analysis before and after UV irradiation

Single-crystal X-ray structure analysis was performed first for an unirradiated **SO** crystal (A), and then UV light was irradiated for 10 seconds before the second measurement (B). Again, UV light was irradiated for 10 seconds, and then the third measurement was performed (C). All measurements were performed at 300 K.

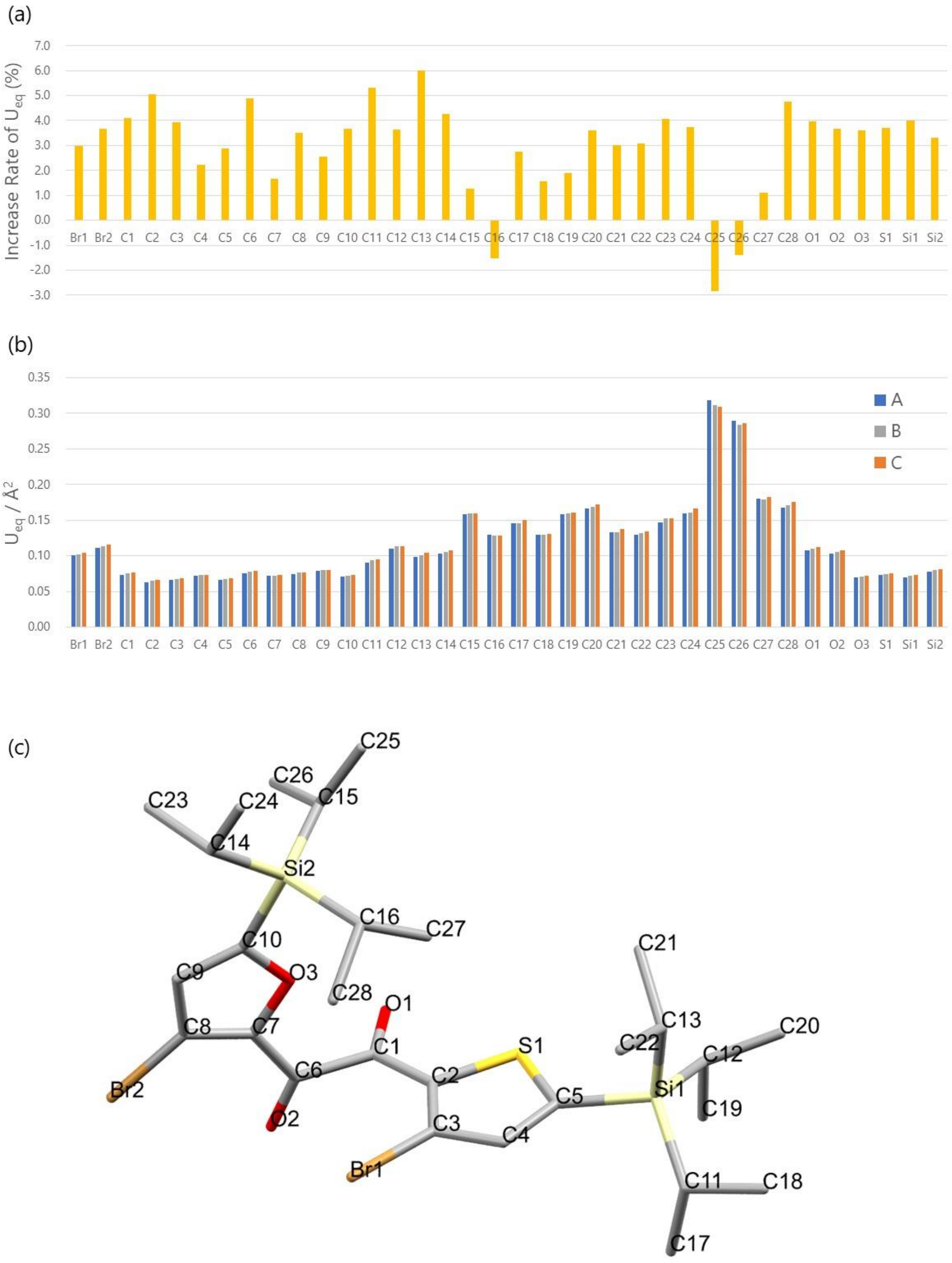

Figure S12. (a) Increase rate of U_{eq} from A to C. (b) U_{eq} of A, B, and C. (c) Crystal structure of C with atom numbering.

Table S2. Crystallographic Data for **SO** (CCDC 2095928, 2189552, 2189553)

Empirical formula	$C_{28}H_{44}Br_2O_3SSi_2$	$C_{28}H_{44}Br_2O_3SSi_2$	$C_{28}H_{44}Br_2O_3SSi_2$
Formula weight	676.69	676.69	676.69
Temperature / K	300	300	300
Crystal system	Monoclinic	Monoclinic	Monoclinic
Space group	$P\ 2_1/n$	$P\ 2_1/n$	$P\ 2_1/n$
a / Å	14.2313(6)	14.2260(6)	14.2236(6)
b / Å	9.2571(5)	9.2521(5)	9.2423(4)
c / Å	26.4870(9)	26.4803(10)	26.4876(9)
α / °	90	90	90
β / °	96.271(4)	96.315(4)	96.355(4)
γ / °	90	90	90
Volume / Å^3	3468.5(3)	3464.2(3)	3460.6(2)
Z	4	4	4
ρ (calcd) / g·cm^{-3}	1.296	1.297	1.299
Crystal size / mm^3	0.8 × 0.3 × 0.2	0.8 × 0.3 × 0.2	0.8 × 0.3 × 0.2
Completeness	99.97 %	99.97%	99.95%
Goodness-of-fit on F^2	1.015	1.009	1.013
Final R indexes [I > =2σ(I)]	R_1 = 0.0545, wR_2 = 0.1558	R_1 = 0.0561, wR_2 = 0.1578	R_1 = 0.0569, wR_2 = 0.1587
Final R indexes [all data]	R_1 = 0.1168, wR_2 = 0.1863	R_1 = 0.1203, wR_2 = 0.1897	R_1 = 0.1248, wR_2 = 0.1940
CCDC number	2252304	2252305	2252306

4. Theoretical study on the conformational dynamics of SO

DFT calculation for the conformers of SO′

Density functional theory (DFT) and time-dependent (TD)-DFT calculations were conducted at the (U)B3LYP-D3/6-311G(d) level of theory using Gaussian 16 program package.[2] All optimized structures were confirmed to be local energy minima without imaginary frequencies.

According to our previous research,[1a] **SO′**, in which the silyl groups of **SO** are replaced with H, has a total eight conformers (each of the four conformers has (*S*) and (*R*)) within a 2 kcal mol^{-1} energy range. Among them, **SO′**-skew is the most stable at the ground state (Figure S13). In contrast, at the T_1 state, the optimized **SO′**-planar is the most stable conformer. Specifically, **SO′**-planar is more stable than **SO′**-skew by 3.9 kcal mol^{-1}. Note that the energy gap become 9.9 kcal mol^{-1} when considering the triisopropylsilyl substituents (i.e., **SO**-planar vs. **SO**-skew) (Figure S14).

To gain insight into the dynamics in excited states, we scanned the coordinates around the thiophene–carbonyl bond starting from the (*S*)-**SO′**-planar in the ground state (Figure S15, the rotation axis is indicated as the green bond). The step of the scan was set to 20°. At each structure, single-point TD-DFT calculations were performed to obtain the S_1 and T_1 energy levels (Figure S15). From the results, we concluded that **SO′**-planar is accessible upon photoexcitation of **SO′**-skew, which corresponds to the conformer in the crystal.

To estimate the activation barrier of the conformational change between **SO′**-skew and **SO′**-planar, we calculated transition-state (TS) geometries in the ground and T_1 states. The obtained geometries were confirmed to be saddle-point structures that have one imaginary frequency. In addition, we displaced the saddle-point geometries along the imaginary frequency and subjected them to structure optimization. As a result, corresponding **SO′**-skew and **SO′**-planar structures in the corresponding electronic states[1a] were obtained. These results indicate that the obtained geometries are the TS for the conformational changes. The estimated Gibbs energy of activation from **SO′**-skew to **SO′**-planar in the T_1 state is quite small (2.4 kcal/mol) while that for the backward conformation change in the ground state (i.e., from **SO′**-planar to **SO′**-skew) is also small (Figure S16).

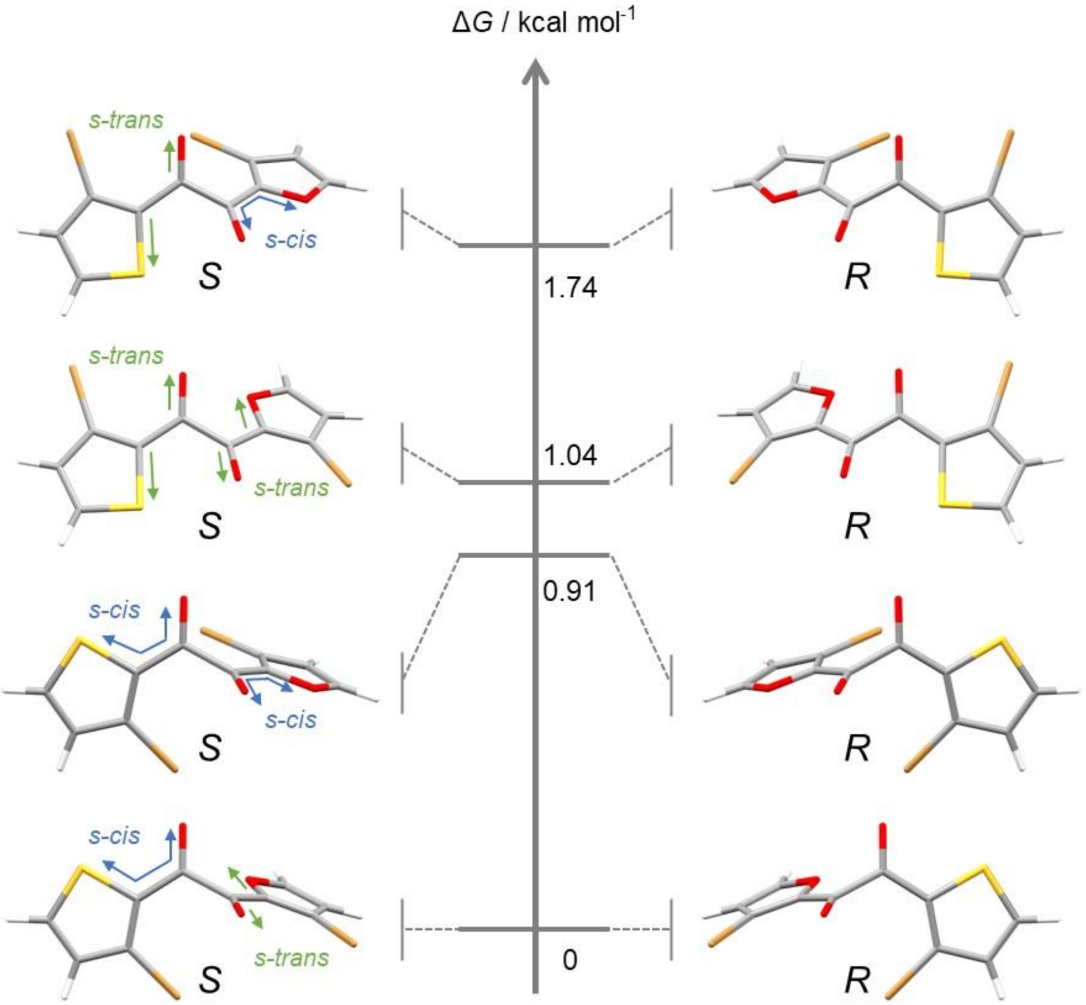

Figure S13. Optimized structures and relative energies of eight conformers of **SO′** in the S_0 state calculated at the B3LYP-D3/6-311G(d) level of theory.

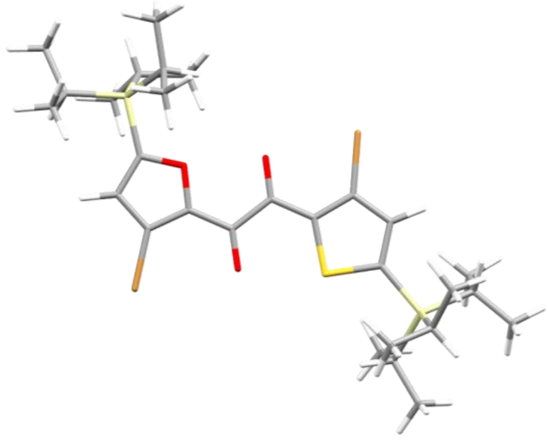

Figure S14. Optimized structure of **SO**-planar in the T_1 state calculated at the UB3LYP-

D3/6-311G(d) level of theory.

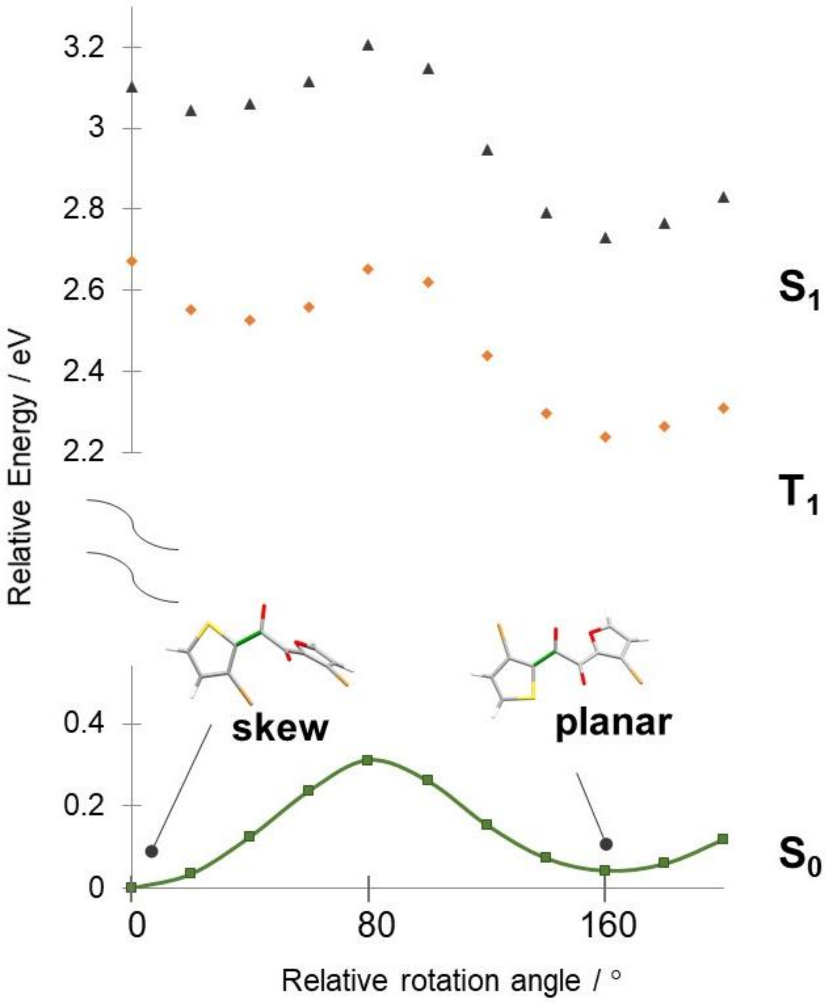

Figure S15. Potential energy curve of isolated (*S*)-**SO′** along the rotation around the green bond calculated at the B3LYP-D3/6-311G(d) level with a step of 20°. Energies for S_1 (black triangle) and T_1 states (orange diamond) were calculated for vertical excitation.

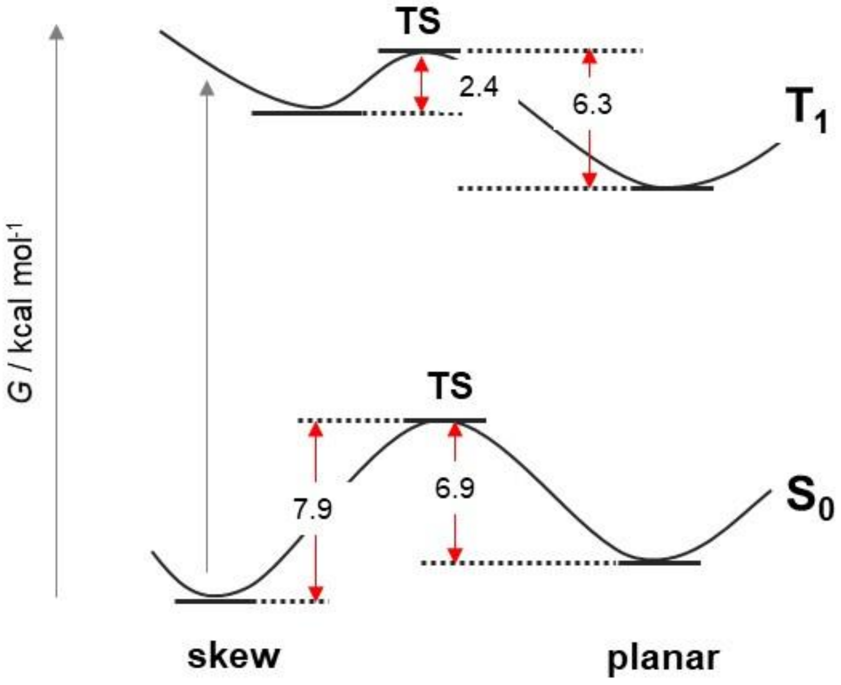

Figure S16. Summary of the calculated Gibbs energies of activation for the conformational change of **SO′**.

Raman spectra of SO crystal

A **SO** crystal and supercooled liquid were placed on a cover strip on the sample stage of home-built Raman microscope, and subjected to the experimental process described in Section 1. The Raman spectra of a liquid and a pristine crystal are shown in Figure S18a and the time-course of the Raman spectra under UV irradiation for a **SO** crystal is shown in Figure S19. The Raman spectrum of the crystal gradually changed and finally matched that of the supercooled liquid, which was prepared by removing solvents from the solution. Although we observed two intense peaks in the range between 1400–1500 cm^{-1}, those peaks are commonly appeared in all the isomers and cannot be used to distinguish isomers, according to the simulated spectra (Figure S18). Nonetheless, the spectrum of the supercooled liquid was more complex than that of the pristine crystal, which suggested the existence of other conformers than the skew conformer (Figure S13 and Figure S18a). In particular, the peak in the range between 1600 and 1700 cm^{-1} became gradually rounded along UV irradiation. According to the simulated Raman spectra (Figure S18b), such spectral changes agree with the occurrence of the planar conformer.”

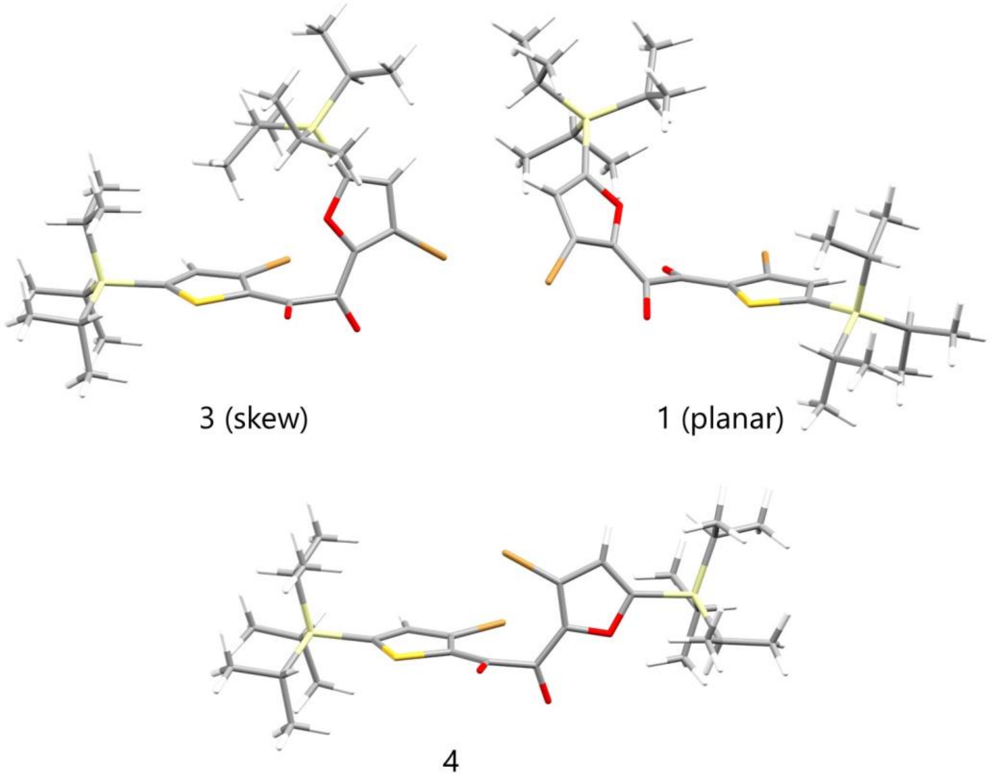

Figure S17. Optimised structures of **SO** conformers in S_0 state calculated at the B3LYP-D3/6-311G(d) level. Skew-B corresponds to the second conformer from the bottom in Figure S13.

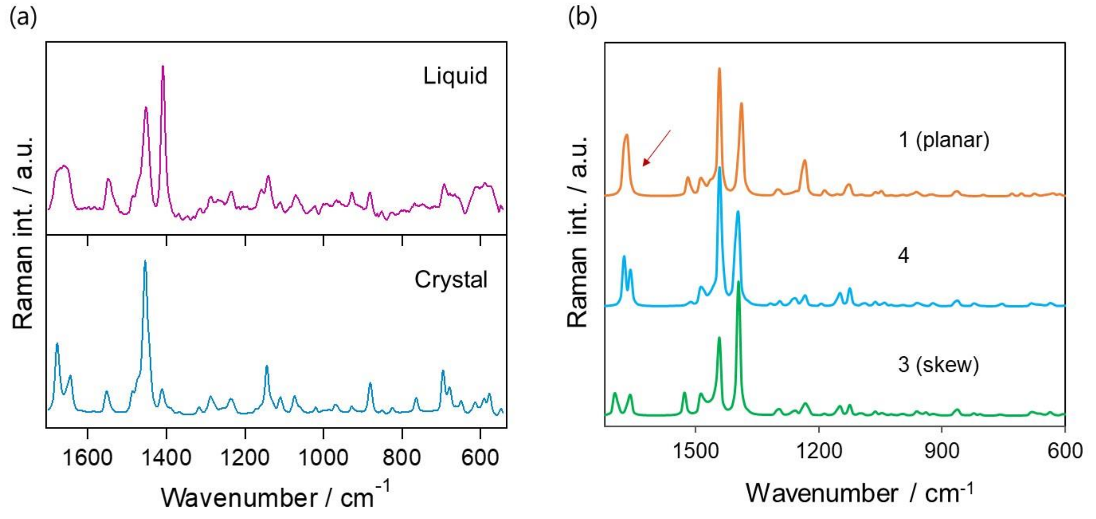

Figure S18. (a) Raman spectra of **SO** single crystal and liquid phases. (b) Simulated Raman spectra of **SO** conformers at the S_0-optimised geometries, calculated at the B3LYP-D3/6-311G(d) level of theory.

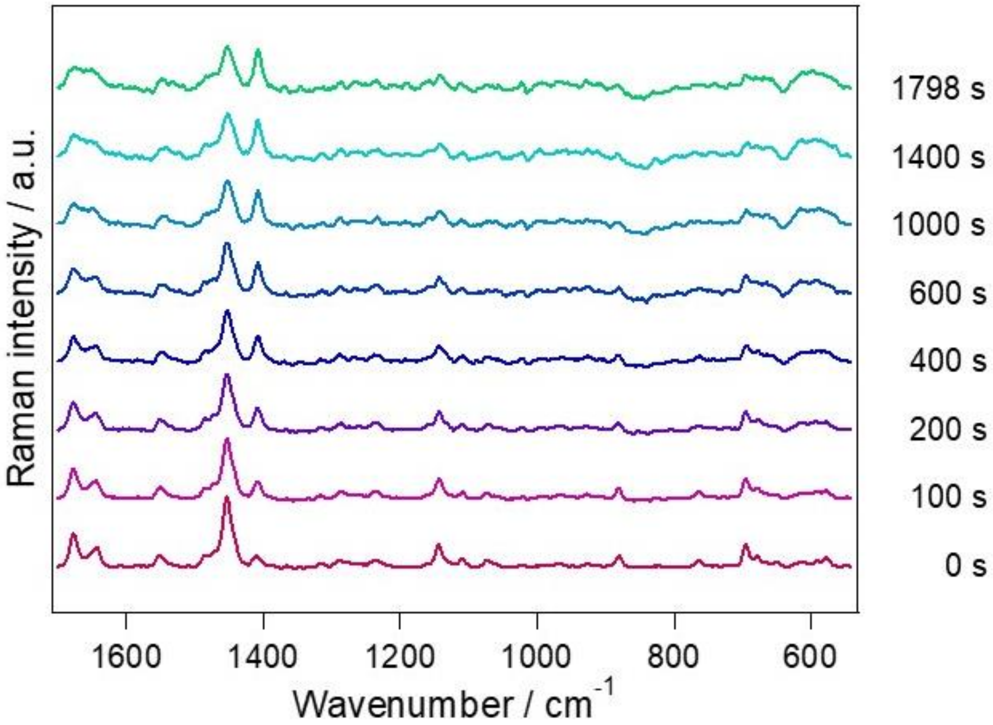

Figure S19. Time-course of the Raman spectra of **SO** crystal under UV irradiation.

DFT calculation for structural relaxation

To examine the structural relaxation upon photoexcitation, we optimized the geometry taken from the crystal structure of **SO**, in the T_1 state. Frequency calculation confirmed that the optimized relaxed skew structure has no imaginary frequency. The skew conformer relaxed with changing the dihedral angle of the dicarbonyl moiety, affording another local minimum structure than the planar conformer (Figure S20). Concomitantly, the silyl moiety moved along the disordered (10–1) plane.

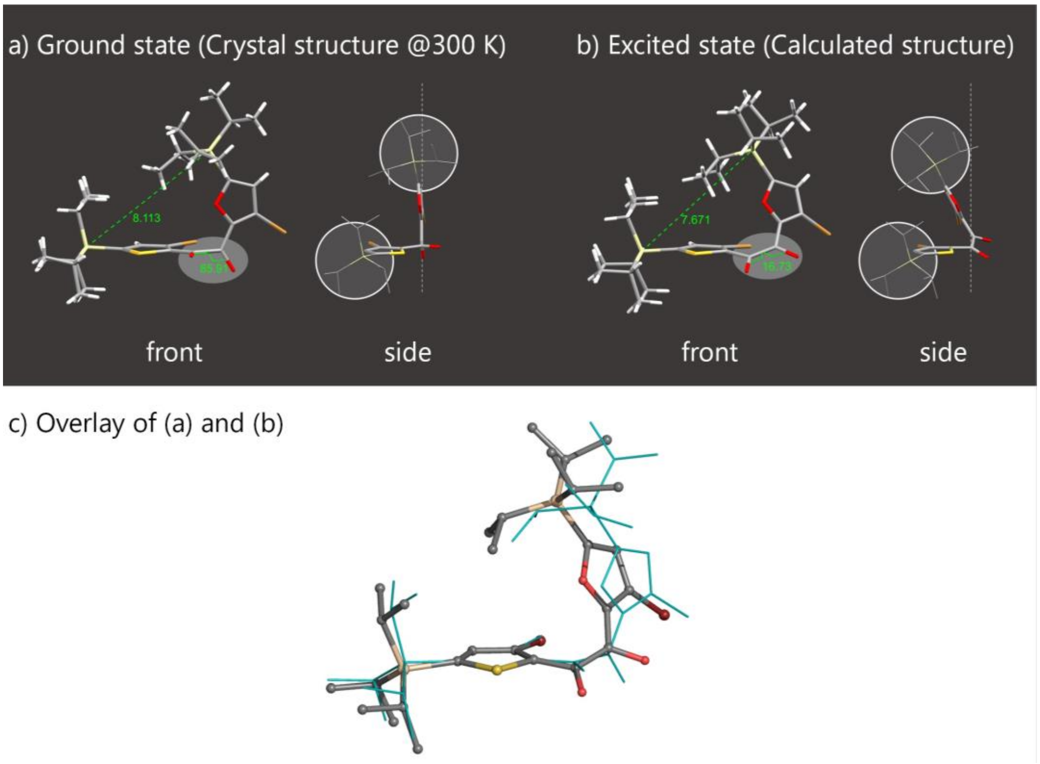

Figure S20. (a) Crystal structure at 300 K and (b) T_1-optimized relaxed skew structure calculated at the UB3LYP-D3/6-311G(d) level of theory. (c) Overlay of the crystal structure (a, cyan line) and the T_1-optimized structure (b, capped stick model).

5. Photoresponses and structures of analogous diketones

Photoresponses of OO and SS

Pristine **OO** powder was obtained by the spontaneous crystallization of the SCL, while pristine **SS** powder was obtained through recrystallization by pouring a $CHCl_3$ solution into MeOH under vigorous stirring. A small amount of the sample was placed on a slide glass under a microscope (instrumentation A). Photographic images were obtained using bright-field and polarizing optical microscopes with a mirror unit (U-MBFL3), after which the mirror unit was switched to U-MWUS to irradiate the sample with UV light. The shape of the **SS** crystal did not change even after more than 11 h of irradiation. In contrast, the **OO** crystal melted completely within 1 min.

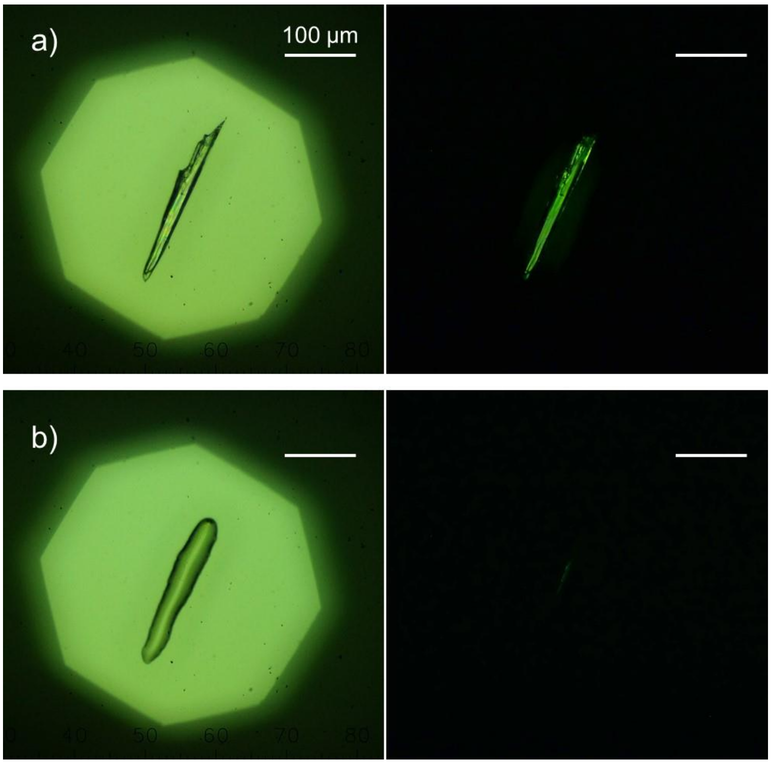

Figure S21. Photographic images of **OO** crystal under (left) bright-field and (right) polarizing optical microscopes with crossed Nichol prisms (a) before irradiation and (b) after continuous UV-light irradiation for 1 min.

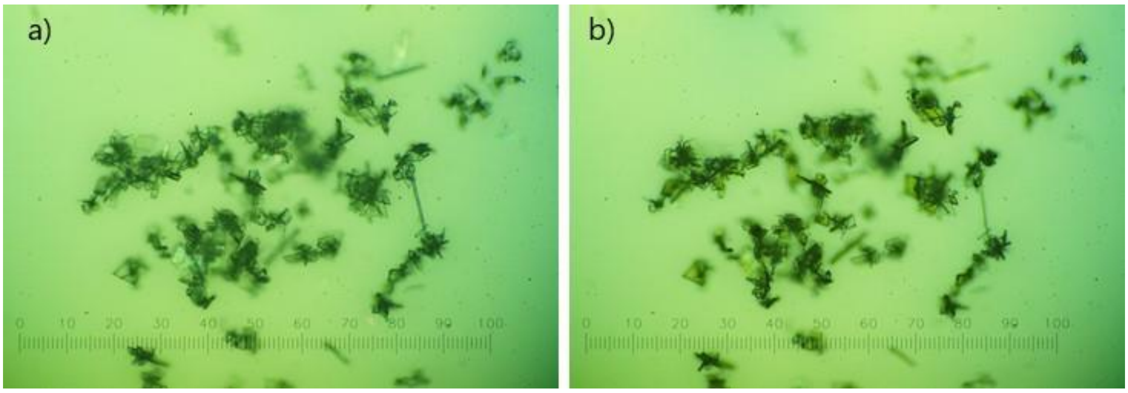

Figure S22. Photographic images of **SS** crystal under bright-field microscope (a) before irradiation and (b) after continuous UV-light irradiation for 11 h. Scale bar = 1 mm.

Comparison of single-crystal X-ray structure analysis of SO, OO, and SS

Crystallographic data of **SS** at 300 K have been reported and deposited to CCDC (1986446) previously.[1a, 1c] Single-crystal of **OO** was obtained by cooling MeOH solution.

Data were successively collected at 123 and 173 K using the same crystal on a Rigaku XtaLAB Synergy-R diffractometer with graphite monochromated MoKα radiation (λ = 0.71075 Å) in the ω-scan mode. The crystal was cooled by a stream of cold N_2 gas. Collection, indexing, peak integration, cell refinement, and scaling of the diffraction data were performed using the CrysAlisPro (Rigaku) software. The structure was solved by direct method (SHELXT 2018) and refined by full-matrix least-square refinement on F^2 (SHELXL2018). The non-hydrogen atoms were refined anisotropically. All hydrogen atoms were placed on the calculated positions and refined using the riding model.

The packing structures (Figures 4b, 4d, and 4e) and strength of the intermolecular interactions in the diketone crystals, described in the main text, are correlated to their molecular conformation. **SO**, **OO**, and **SS** exhibited skewness with respect to the dicarbonyl moiety. However, their conformations differ with respect to the twist angle between one of the aromatic rings and the adjacent carbonyl group: C–S (or C–O) and C=O are *s-cis* in the case of **SS** and *s-trans* in the cases of **SO** and **OO** (Figure 4a and Figure S23). Different conformations in the crystal can be due to the relative stability of the two skew conformers, which is dictated by a steric effect. Thiophene and furan are geometrically different due to the different bond length/angle around the heteroatom. In particular, the exocyclic bond angles of 2- and 3-positions are approximately 71.6° and 80.4° for thiophene and furan, respectively. Consequently, in the *s-trans* arrangement, the carbonyl oxygen becomes closer to the bromine atoms in **SS** (thiophene) than in **SO** and **OO** (furan); the calculated Br–O distance is shorter than the sum of their van der Waals radii (3.35 Å) in **SS′** (Figure S24). This steric hindrance destabilises the s-cis arrangement of **SS**.

As a result of these *s-cis*/*s-trans* conformation differences, **SS** has a rod-like shape while **SO** and **OO** are spherical. The spherical shape reduces the specific surface area and packing density, thereby decreasing the strength of the van der Waals interactions.

These conclusions were confirmed quantitatively based on a Hirshfeld surface analysis (see below, Figure S25)[3] and partly explain why the enthalpies of fusion (and hence the intermolecular interactions) of the **SO** and **OO** crystals are smaller than that of the **SS** crystal. In addition, the two silyl groups of **SO** and **OO** are on one side of the molecule while those of **SS** are located at the two ends of its rod-like structure. This asymmetrical distribution of the C–H-bond-rich surface may be critical to the formation of the characteristic packing structure of **SO** and **OO**. Moreover, conformational changes require the rod-like structure of **SS** to be bent against the stronger intermolecular interactions between the core skeletons. Therefore, it can be assumed that **SS** requires more energy for relaxation/isomerization than do **SO** and **OO**.

Table S3. Crystallographic Data for **OO** (CCDC 2189550–2189551)

Empirical formula	$C_{28}H_{44}Br_2O_4Si_2$	$C_{28}H_{44}Br_2O_3SSi_2$
Formula weight	660.63	660.63
Temperature / K	123	173
Crystal system	Monoclinic	Monoclinic
Space group	$P2_1/c$	$P2_1/c$
a / Å	15.3021(5)	15.3539(4)
b / Å	14.6862(4)	14.7515(3)
c / Å	29.0678(9)	29.2022(8)
α / °	90	90
β / °	98.666(3)	98.737(2)
γ / °	90	90
Volume / $Å^3$	6457.8(3)	6537.3(3)
Z	8	8
ρ (calcd) / g·cm^{-3}	1.359	1.342
Crystal size / mm^3	0.25 × 0.2 × 0.15	0.25 × 0.2 × 0.15
Completeness	99.90%	99.96%
Goodness-of-fit on F^2	1.163	1.131
Final R indexes [$I >= 2\sigma(I)$]	$R_1 = 0.0768$, w$R_2 = 0.1723$	$R_1 = 0.0782$, w$R_2 = 0.1828$
Final R indexes [all data]	$R_1 = 0.1169$, w$R_2 = 0.1841$	$R_1 = 0.1336$, w$R_2 = 0.1998$
CCDC number	2189550	2189551

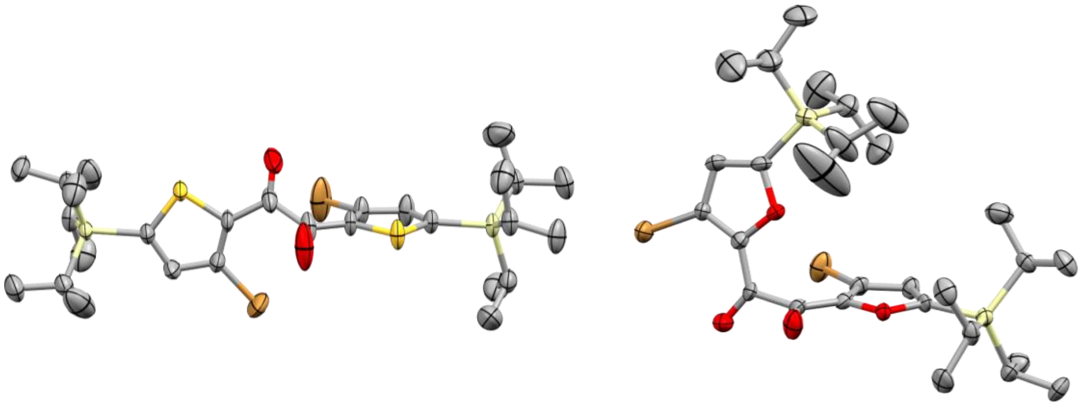

Figure S23. ORTEP drawing **SS** (left) at 300 K and **OO** (right) at 173 K. Thermal ellipsoids are set at 50 % probability level.

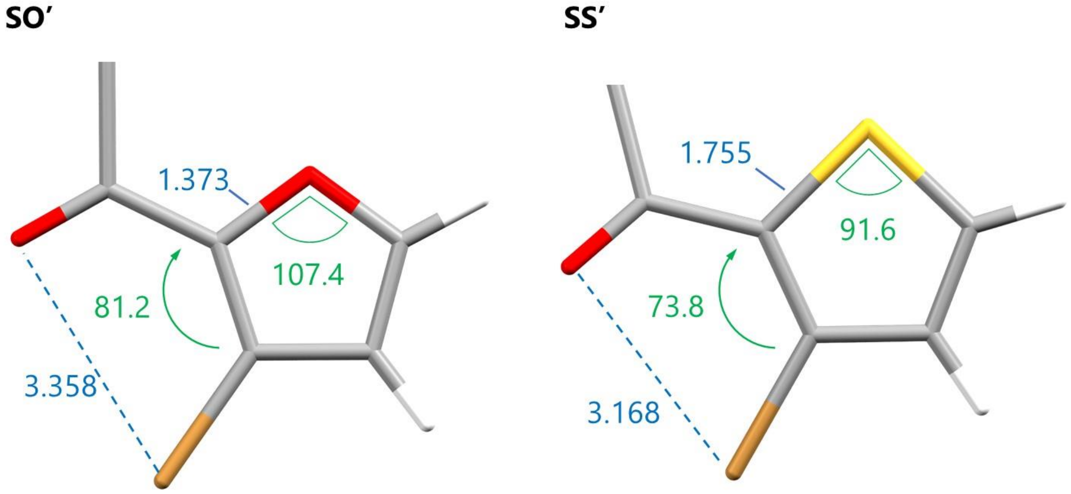

Figure S24. The optimised geometries, selected bond lengths (Å), distances (Å), and angles (°) of the *s-trans* conformers for SO′ and SS′ in the S_0 state calculated at the B3LYP-D3/6-311G(d) level of theory (only half of the molecules was shown).

Hirshfeld surface analyses of SO, OO, and SS crystals

Hirshfeld surface analysis provides a way to view molecules as "organic wholes".[3a] To investigate the intermolecular interactions visually and quantitatively, Hirshfeld surfaces of **SO**, **OO**, and **SS** in the crystal structure at 123 K were mapped with normalized contact distance d_{norm} with a fixed color scale of –0.3 (red) to 1.7 (blue) using CrystalExplorer21 software (Figure S25).[3b] d_{norm} is defined as follows:

$$d_{norm} = \frac{d_i - r_{vdW}}{r_{vdW}} + \frac{d_e - r_{vdW}}{r_{vdW}}$$

where d_i is the distance from the Hirshfeld surface to the nearest nucleus inside the surface, d_e is the corresponding distance to the nearest nucleus outside the surface, and r_{vdW} is the van der Waals radius of the atoms. The positive (blue) d_{norm} indicates a distance larger than van der Waals contact. As a whole, the surface of **SO** and **OO** are bluer than that of **SS**, and the mean d_{norm} are larger for **SO** and **OO** than **SS**. In addition, the surface area is larger for **SS** than **SO** and **OO** (609 Å^2 vs. 570 and 549 Å^2). The asphericity is also larger for **SS** than **SO** and **OO**. These results suggest that the intermolecular interactions are weaker in **SO** and **OO** than **SS**.

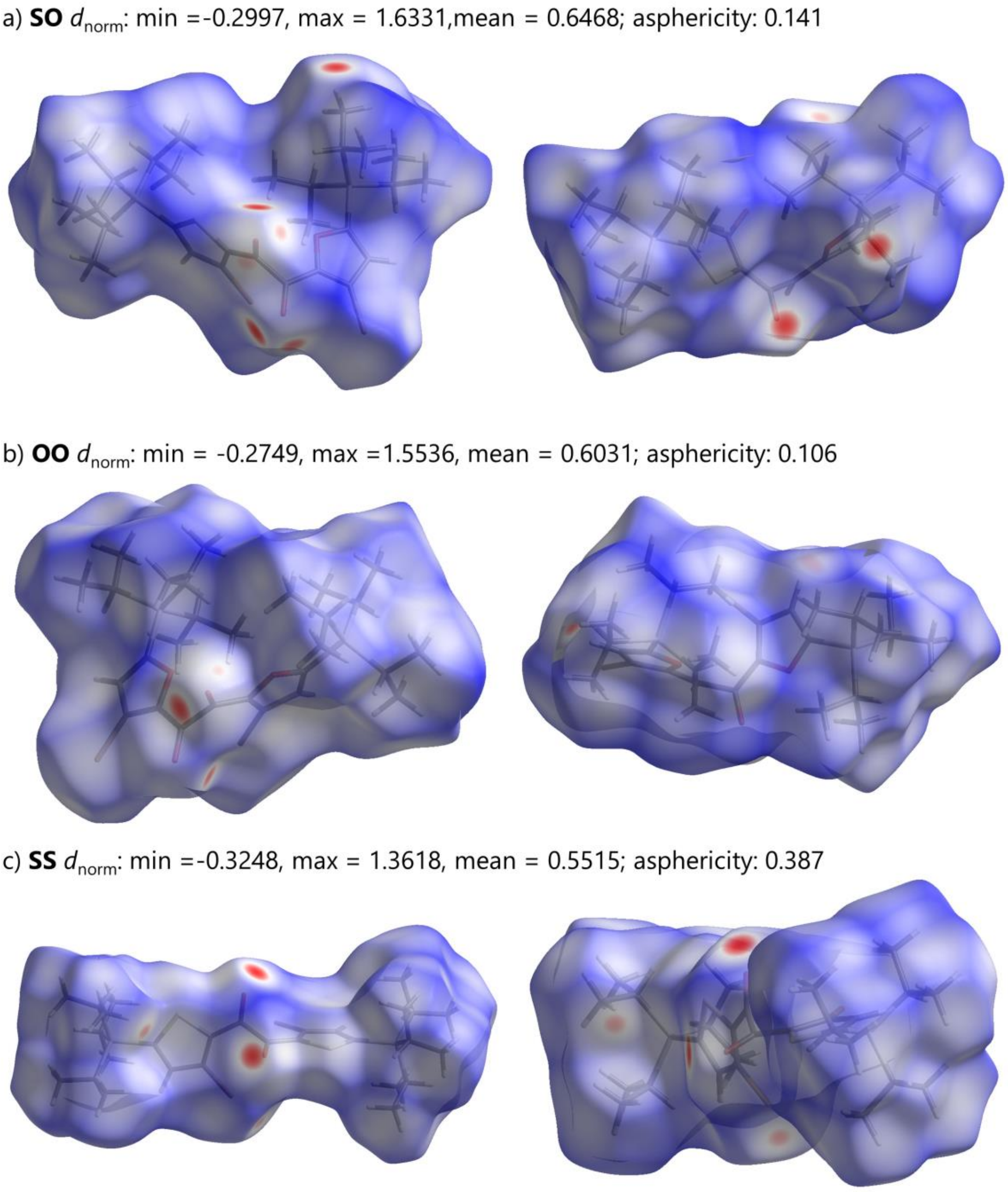

Figure S25. Hirshfeld surface of (a) **SO**, (b) **OO**, and (c) **SS** mapped with d_{norm} (mapped over a fixed color scale of –0.3 (red) to 1.7 (blue)). The surfaces as well as the minima, maxima, and mean values of d_{norm}, and asphericity were obtained by CrystalExplorer21.

Differential scanning calorimetry (DSC)

DSC was performed with a Hitachi NEXTA DSC200 under a flow of nitrogen. Solid samples of **SO** and **OO** were obtained by spontaneous crystallization of the SCL, while that of **SS** was obtained by pouring $CHCl_3$ solution into MeOH with vigorous stirring. The enthalpies of fusion (ΔH_m) of **SO**, **OO**, and **SS** were estimated to be 22, 29, and 40 kJ/mol, respectively, from the area of the endothermic melting peaks appeared in the heating traces. The entropies of fusion (ΔS_m) were estimated according to the formula $\Delta H_m = T_m \Delta S_m$, where T_m is the melting point. Gibbs free energies for the crystal-to-SCL transition at 300 K (ΔG) were estimated to be 2.2, 2.7, and 12 kJ/mol for **SO**, **OO**, and **SS**, respectively, according to the formula $\Delta G = \Delta H_m - T \Delta S_m$.

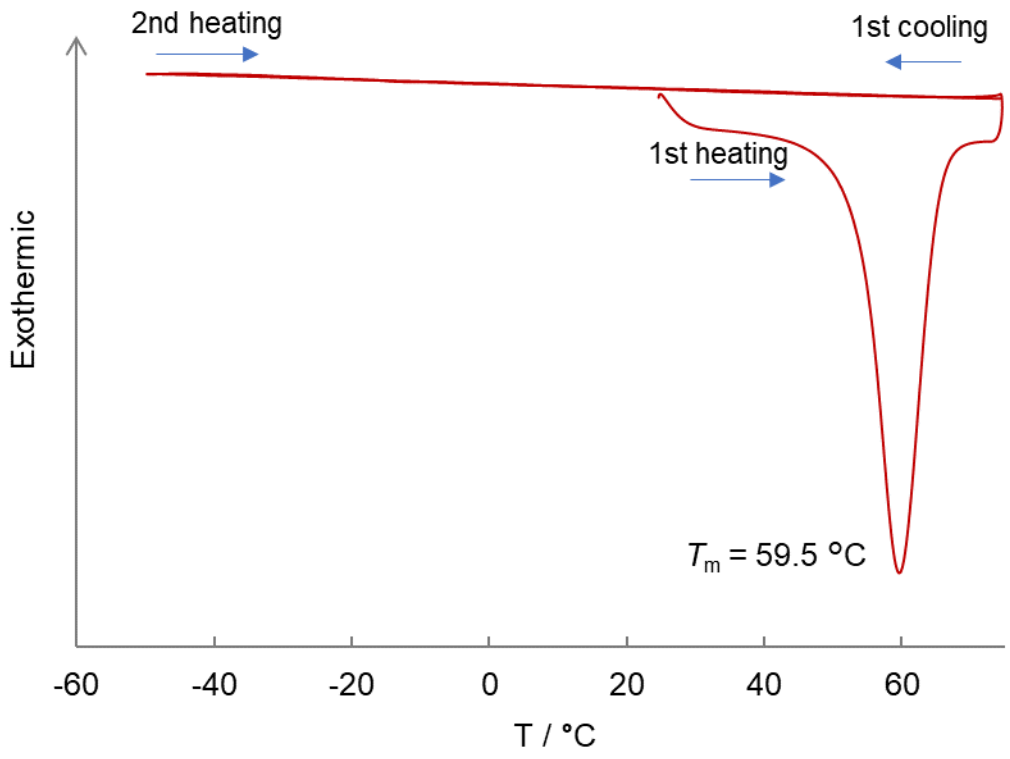

Figure S26. DSC thermogram of **SO** during heating/cooling cycles at 10 °C min^{-1} for the 1st heating and at 0.3 °C min^{-1} for the 1st cooling and 2nd heating processes, under a flow of N_2.

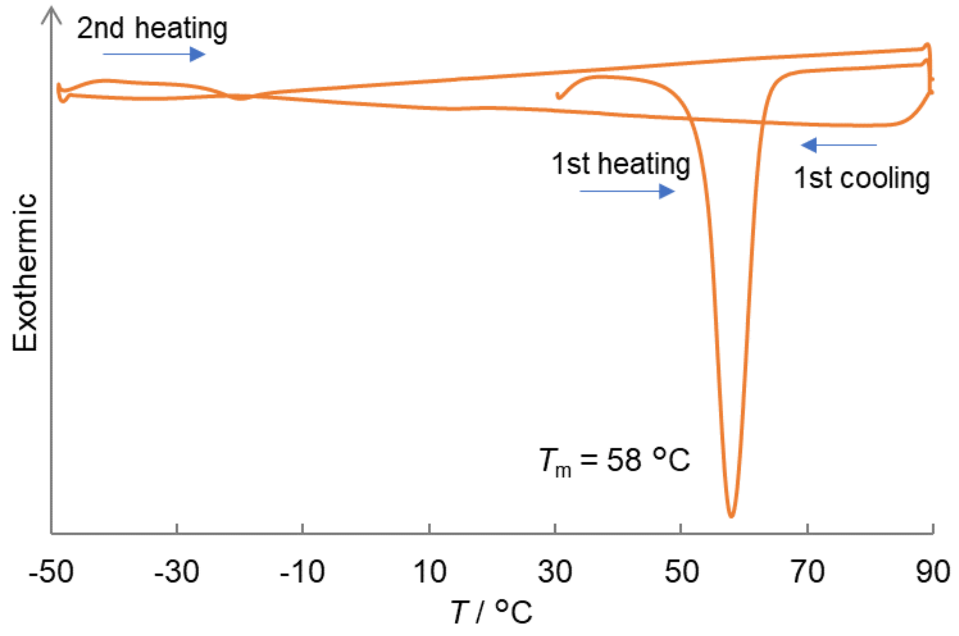

Figure S27. DSC thermogram of **OO** solid during heating/cooling cycles at 10 °C min^{-1} under a flow of N_2.

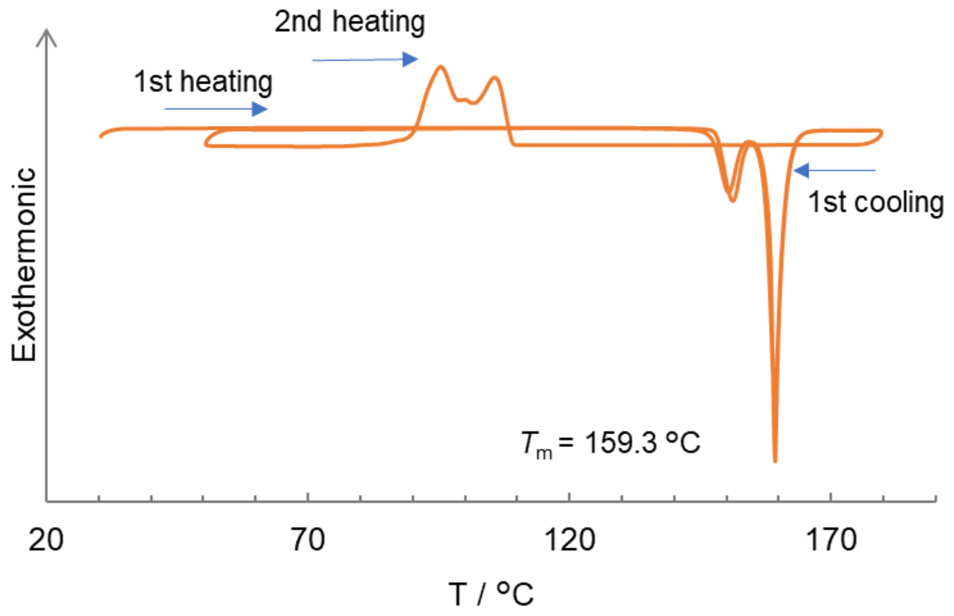

Figure S28. DSC thermogram of solid **SS** during heating/cooling cycles at 10 °C min^{-1} under a flow of N_2.

DFT calculation

DFT calculations were conducted at the (U)B3LYP-D3/6-311G(d) level of theory using Gaussian 16 program package.[2] All optimized structures were confirmed to be local energy minima without imaginary frequencies. The skew and planar conformers of **OO** **SS** were optimized in the T_1 state. Frequency calculation confirmed that all the optimized structures have no imaginary frequency.

The calculations suggest that the planar conformers of **OO** and **SS** are more stable than the skew conformer in the T_1 states (Table S4).

Table S4. The Gibbs Free Energies (ΔG) of the Planar Conformer Relative to the (Relaxed) Skew Conformer at the T_1-Optimized Geometries

Diketone	ΔG / kcal mol^{-1} [a]
**SO**	–9.89
**OO**	–8.35
**SS**	–6.38

[a] The energies include thermal correction at 298.150 K.

6. References

1. (a) M. Komura, T. Ogawa, Y. Tani, *Chem. Sci.*, **2021**, *12*, 14363–14368. (b) Y. Tani, M. Komura, T. Ogawa, *Chem. Commun.* **2020**, *56*, 6810–6813. (c) Y. Tani, M. Terasaki, M. Komura, T. Ogawa, *J. Mater. Chem. C* **2019**, *7*, 11926–11931.
2. M. J. Frisch, G. W. Trucks, H. B. Schlegel, G. E. Scuseria, M. A. Robb, J. R. Cheeseman, G. Scalmani, V. Barone, G. A. Petersson, H. Nakatsuji, X. Li, M. Caricato, A. V. Marenich, J. Bloino, B. G. Janesko, R. Gomperts, B. Mennucci, H. P. Hratchian, J. V. Ortiz, A. F. Izmaylov, J. L. Sonnenberg, D. Williams-Young, F. Ding, F. Lipparini, F. Egidi, J. Goings, B. Peng, A. Petrone, T. Henderson, D. Ranasinghe, V. G. Zakrzewski, J. Gao, N. Rega, G. Zheng, W. Liang, M. Hada, M. Ehara, K. Toyota, R. Fukuda, J. Hasegawa, M. Ishida, T. Nakajima, Y. Honda, O. Kitao, H. Nakai, T. Vreven, K. Throssell, J. A. Montgomery, Jr., J. E. Peralta, F. Ogliaro, M. J. Bearpark, J. J. Heyd, E. N. Brothers, K. N. Kudin, V. N. Staroverov, T. A. Keith, R. Kobayashi, J. Normand, K. Raghavachari, A. P. Rendell, J. C. Burant, S. S. Iyengar, J. Tomasi, M. Cossi, J. M. Millam, M. Klene, C. Adamo, R. Cammi, J. W. Ochterski, R. L. Martin, K. Morokuma, O. Farkas, J. B. Foresman, and D. J. Fox, , *Gaussian 16*

Rev. C.02; Wallingford, CT, 2019.

3. (a) M. A. Spackman, D. Jayatilaka, *CrystEngComm* **2009**, *11*, 19–32. (b) P. R. Spackman, M. J. Turner, J. J. McKinnon, S. K. Wolff, D. J. Grimwood, D. Jayatilaka, M. A. Spackman, *J. Appl. Cryst.* **2021**, *54*, 1006–1011.
